# General skin and nasal decolonization with octenisan® set before and after elective orthopedic surgery in selected patients at elevated risk for revision surgery and surgical site infections—a single-center, unblinded, superiority, randomized controlled trial (BALGDEC trial)

**DOI:** 10.1186/s13063-024-08173-y

**Published:** 2024-07-08

**Authors:** Ines Unterfrauner, Nadja Bragatto-Hess, Thorsten Studhalter, Mazda Farshad, Ilker Uçkay

**Affiliations:** 1 Orthopedic Surgery, Balgrist University Hospital, University of Zurich, Forchstrasse 340, 8008 Zurich, Switzerland; 2 Infection Control, Balgrist University Hospital, University of Zurich, Forchstrasse 340, 8008 Zurich, Switzerland; 3 Unit for Clinical and Applied Research, Balgrist University Hospital, University of Zurich, Forchstrasse 340, 8008 Zurich, Switzerland; 4 Medical Direction, Balgrist University Hospital, University of Zurich, Forchstrasse 340, Zurich, 8008 Switzerland

**Keywords:** Elective orthopedic surgery, High risk patients, Body decolonization, Surgical site infections, Octenidine, Questionnaire, Wound problems, Randomized controlled trial

## Abstract

**Background:**

The preoperative body surface and nasal decolonization may reduce the risk of surgical site infections (SSI) but yields conflicting results in the current orthopedic literature.

**Methods:**

We perform a single-center, randomized-controlled, superiority trial in favor of the preoperative decolonization using a commercial product (octenidine® set). We will randomize a total number of 1000 adult elective orthopedic patients with a high risk for SSI and/or wound complications (age ≥ 80 years, chronic immune-suppression, American Society of Anesthesiologists score 3–4 points) between a decolonization (octenisan® wash lotion 1 × per day and octenisan® md nasal gel 2–3 × per day; during 5 days) and no decolonization. Decolonized patients will additionally fill a questionnaire regarding the practical difficulties, the completeness, and the adverse events of decolonization. The primary outcomes are SSI and revision surgeries for postoperative wound problems until 6 weeks postoperatively (or 1 year for surgeries with implants or bone). Secondary outcomes are unplanned revision surgeries for non-infectious problems and all adverse events. With 95% event-free surgeries in the decolonization arm versus 90% in the control arm, we formally need 2 × 474 elective orthopedic surgeries included during 2 years.

**Discussion:**

In selected adult orthopedic patients with a high risk for SSI, the presurgical decolonization may reduce postoperative wound problems, including SSI.

**Trial registration:**

ClinicalTrial.gov NCT05647252. Registered on 9 December 2022.

Protocol version: 2 (5 December 2022).

**Supplementary Information:**

The online version contains supplementary material available at 10.1186/s13063-024-08173-y.

## Introduction

### Background and rational

The bacterial skin (and nasal) colonization due to *Staphylococcus aureus* and with other skin commensals (coagulase-negative staphylococci, micrococci, cutibacteria, corynebacteria) is very probably the main source of intraoperatively acquired surgical site infections (SSI) after elective orthopedic, especially in the present of a recently introduced implants. This chronic colonization is very tenacious and can only be transiently reduced by pre-surgical scrubbing, pre-incisional hand disinfection, or preoperative decolonization [1]. The general decolonization of the human body surface and the nose before elective surgery [1–3] is recommended by the World Health Organization (WHO) [4]. It aims to reduce the risk of superficial and/or deep surgical site infections (SSI) and associated wound problems. Even if there are promising, and pioneering, studies on favor of a general or targeted preoperative decolonization in the beginning of the new century, the recent years also came up with well-conducted RCTs advocating against a benefit of decolonization, especially not for every orthopedic patient [5–8]. In summary, the decolonization is considered as evidence-based according to the majority of the initial “before-and-after studies” but remains inconclusive in later randomized controlled trials (RCTs) [5, 6]. The pre-surgical decolonization is only one of all preventive measures embedded in an entire bundle of combined efforts to prevent SSIs. Its individual power is limited for young, healthy individuals or regarding SSIs caused by pathogens from internal body sites (intestinal, urinary, gynecologic regions). It is even astonishing that a single preoperative procedure such as the decolonization would really reverse the ultimate fate of SSI by it alone [1]. Likewise, the presurgical action may not work to prevent SSIs that are acquired postoperatively on the ward [9, 10] or in settings with a low volume of surgical experience [1]. Hence, in trials including the entire orthopedic population without stratifications, the beneficial effect of decolonization may disappear, and the procedure may become costly and cumbersome for patients with low inherent SSI risks.

Moreover, the ideal modalities of the decolonization procedures, agents, duration, and timing still remain unknown. Equally, we ignore if we should administer this procedure for every orthopedic patient or primarily to selected strata of patients. In the orthopedic field, this decolonization is likely to be more effective in patients with a proven body surface carriage of *Staphylococcus aureus* [2, 3] but theoretically acts on all bacteria that are accessible to topical agents [7–10]. Many SSIs in elective orthopedic surgery, especially in implant-related surgery, are due to coagulase-negative staphylococci (CoNS) [11, 12]. The hallmark of the CoNS group is *S. epidermidis* [13] with approximatively 70% usual antibiotic resistance (in Switzerland) to standard perioperative prophylactic agents such as cefuroxime [11]. As *S. epidermidis* is part of the normal human flora [13], a presurgical screening is not feasible, because everyone is colonized with CoNS in general and with *S. epidermidis* in particular [13]. Unsurprisingly, with so much natural methicillin resistance, the prophylaxis-resistant part of all SSIs is 30–50% [14, 15].

In our single-center, unblinded, prospective-randomized, superiority trial over a period of 2 years, we target on an orthopedic patient population with an inherent elevated risk for revision surgery and SSIs: elderly patients, immune-suppressed individuals, and those with many co-morbidities [1, 11, 16, 17]. We will use an existing “set” manufactured by Schülke & Mayr [7, 8]. The “set” is on the Swiss market since 2016. The distribution of octenisan® wash lotion and octenisan® nasal gel in the form of a set (octenisan® set) facilitates the application and the compliance efforts for our study. Academically, we will gain more insight in the performance of the decolonization for a patient population at high risk of infectious complications (SSIs and wound problems), for whom every preventive effort is of utmost importance [1, 17–21].

## Methods and materials

### Study setting

The Balgrist University Hospital in Zurich is a tertiary referral center for orthopedic surgery and affiliated to the University of Zurich. It has a multi-disciplinary team composed of orthopedic surgeons, internists, infection control nurses, and infectious diseases physicians who are all specialized in orthopedic infections. Moreover, this team is accompanied by the Unit for Clinical and Applied Research (UCAR) with experience in investigative designs. The UCAR engages 4 study nurses and 3 research assistants specialized in clinical trials.

## The decolonization set—product

Schülke & Mayr will donate 550 decolonization sets for free use for the BALGDEC trial.

We will use these prefabricated set [7, 8] that contain patient’s information leaflets available in German, English, French, and Italian languages. The active ingredient contained in both products (octenisan® wash lotion and octenisan® md nasal gel; both products combined in octenisan® set) [8] (no. article 11636528, EAN 4032651979264) is octenidine dihydrochloride. One set would cost 22.25 Swiss Francs on the market [8]. The wash lotion is applied once a day and rinsed with water after application. The nasal gel is applied 2–3 times per day [7, 8]. The frequency choice is at the discretion of the patient, as no differences in outcome and efficacy between 2 or 3 times are known in the literature. We keep this free choice, equally permitted by the manufacturer, in the protocol without artificially fixing a firm number of daily applications. The sets can be stored at ambient temperature for several months, as indicated on the packaging. The study sets will be locked in the office of the infection control nurses and in the PI’s (principal investigator) office. There is no public access to these offices without individual keys.

## Study objectives

We aim to reduce the incidence of SSI (and other unplanned postoperative wound revisions) in adult orthopedic patients with an elevated risk for SSI. We equally investigate the safety of the presurgical decolonization, and the difficulties of its application, in daily clinical life.

## Study criteria, definitions and study outcomes

**Fig. 1:**
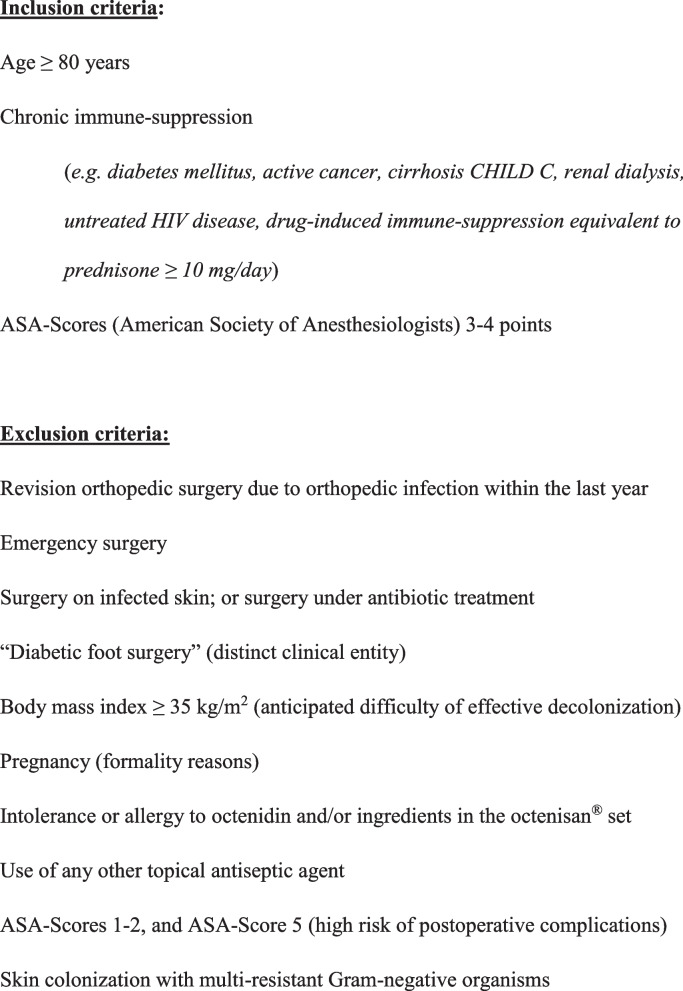
Study criteria

**Fig. 2:**
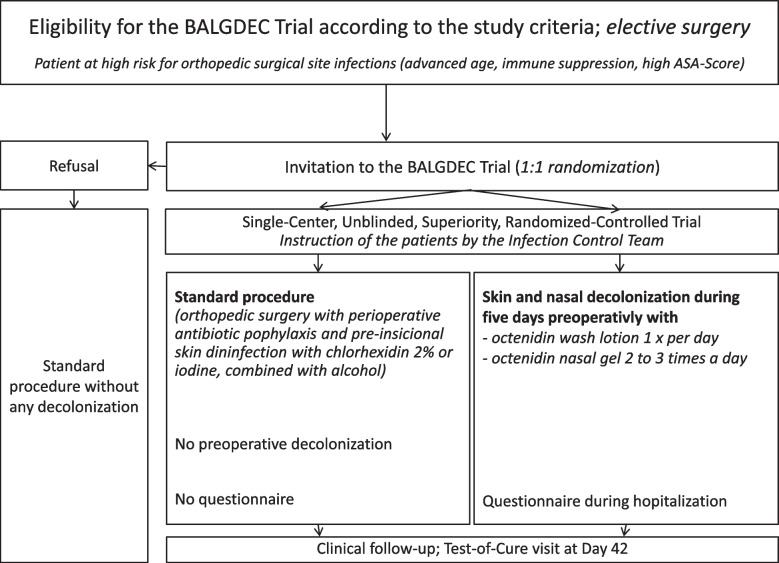
Main study flowchart

Tables 1 and 2 present key definitions and the outcomes of the trial. Only adult, elective orthopedic surgery patients, with a focus on high risk for revision and/or SSI, will be included. This particular patient population presents the following: chronic immune suppressions of any type, patients with American Society of Anesthesiologists (ASA) scores of 3–4 points or with an age of ≥ 80 years, independently of the presence of an orthopedic implant. Fig. 1 presents the study criteria, and Fig. 2 presents the study flowchart. We define SSI as the microbiological evidence of the same bacteria in at least two intraoperative tissue samples together with radiological (osteitis, collections, inflammation) and/or clinical evidence of infection (pus, discharge, sinus tracts, rubor, calor, pain). The presence of a histological proof is facultative. Postoperative wound problems are any unexpected problems that persist, or re-emerge, after 10 days following the elective orthopedic interventions. We define implants as any foreign material, except for allografts, wires, or fixator pins. “Remission” is the absence of clinical, and/or radiological, and/or laboratory signs of infection after a minimal follow-up time of 6 weeks for soft-tissue surgery or 1 year for implant-related and/or bone surgery.

**Table 1:** Key study definitions

Decolonization: Skin and nasal decolonization during five days preoperatively with octenidine wash lotion 1 × per day plus octenidine nasal gel 2 to 3 times a day (at the discretion of the patient) Regular change of underwear and bed linen
Surgical site infection (SSI): Postoperative infection at the operated body site, defined by clinical signs, e.g., pus, fever, rubor, and calor, together with the identification of the same pathogen(s) in at least two intraoperative microbiological samples. The histopathology is facultative for the diagnosis of infection
Non-infected wound problems: Any surgical wound problem leading to a prolongation of the hospital stay or to new therapeutic measures besides the regular wound dressings (e.g., revision surgery, negative-vacuum therapy)
Remission: Absence of clinical, anamnestic, radiological, or laboratory signs for infection at the test-of-cure visit (or at 1 year’s follow-up in case of an implant and bone surgery)

**Table 2:** Outcome parameters and assessments of the randomized trial

Primary outcome (composite outcome) - Remission (and inversely superficial or deep-space SSI and revision surgery for postoperative wound problems) at 6 weeks (and/or a 1 year for surgeries with implant)
Secondary outcomes: - Unplanned revision surgery for non-infection problems in same time period - All adverse events during decolonization and hospitalization for surgery - Subjective opinion on the decolonization (only for patients being decolonized; using a questionnaire)

Assessment of outcomes: prospective assessment by the infection control team during hospitalization. Retrospective assessment by study nurses and surgeons during the surgical controls after hospitalization. These surgical controls are regularly scheduled at 6 weeks and 1 year postoperatively, independently of our study

## Interventions and study conduct

The BALGDEC trial is a single-center trial, starting on 27 February 2023. The operational study team will screen eligible patients during the presurgical orthopedic and anesthesiologic consultations, which usually take place between 3 and 10 days before the scheduled surgery.

All clinicians may screen for eligible patients and inform them about the BALGDEC trial. However, only the infection control nurses and the infectious diseases physician will provide specific instructions regarding the decolonization measure. We will randomize the included patients (1:1) to the “decolonization set” or no medical decolonization. The study team will hand over the set at enrolment, together with an in-house questionnaire. The randomization procedure uses prefabricated cards and is performed by a study nurse who is not involved in the enrolment process (central telephone). These cards are pre-printed and kept locked within envelopes in a dedicated wardrobe in the separate office of the study nurses, already before the inclusion of the first patient. The dedicated study nurse (or her replacement) receives a phone call of the infection control team who includes the patients. At this time, she pulls a card and randomizes. The cards of the included patients are attached to their consent form. Every card is used only once. Hence, the including person and the randomizing person are always separated and do not work on a common electronic database. There will be no blinding and no placebos. Each surgery counts as an independent event. During the 2 years that we plan for this trial, one patient can participate several times as long as his/her orthopedic surgeries are independent from each other.

### Decolonization procedure

The duration of the pre-surgical decolonization is 5 days. When this period is too short (because of the later anticipation of the surgery slot by 1 to 2 days for strictly organizational reasons), the decolonization may also start 3 days before surgery and be continued until day 2 post-surgery. For hospitalized patients, or patients in elderly homes, the nurses might apply the products. Likewise, for patients with cognitive disabilities, an instructed family member can also decolonize. During decolonization, patients shall change bed linens and the underwear every day. They must not share towels, clothes, and other textiles with family members or pets. They must not use other topical antiseptic products on the skin, body lotions, or moisturizers. However, the study patients are allowed to use habitual parfums. The patients will return the empty/used sets and answer to a questionnaire that we handed over. The study team will recuperate the questionnaire during hospitalization and fill in lacking responses bedside and together with the patient. We aim a return rate of the questionnaires of at least 90%. In the BALGDEC study, we purposely renounce on routine microbiological assessments of skin colonization, because most SSIs are due to usual skin commensals. We screen patients only for the carriage of antibiotic-resistant pathogens, if they correspond to recommendations of the Swiss Infection Control Guidance (www.swissnoso.ch). However, we may also analyse this extra-protocolled additional clinical data.

### Accountability of the decolonization set

We recuperate the empty packages of the used decolonization sets from the decolonized patients shortly after surgery. This process will be documented (accountability log). This log also serves to record any damages of the set. The empty sets will be archived, during the trial, in a side room annex to the infection control nurses and destroyed after the end of the trial. If the returned packages are not empty, we will not use the content for other patients.

## Procedures at each visit

At enrollment (visit 1/day 1), the infection control team informs and recruits the patients. The study nurse randomizes the included patients at a ratio of 1:1 into the investigational group (decolonization) and the control group (no decolonization). The infection control team will instruct the correct decolonization procedure and distribute the questionnaire to the decolonization group. The study period includes the following study visits (Table 3):

**Table 3:** Study assessments during the visits

Study period	Screening/baseline*	Visit 1* Enrolment	Visit 2* End-of-treatment visit	Visit 3 Test-of-cure visit	Follow-up for surgeries with implants
**Time**	Day − 30 to 0	Enrolment	Surgery day or 1–2 days after surgery	6 weeks (± 14 days)	1 year (± 2 months)
Inclusion/exclusion criteria	X	X			
Informed consent	X	X			
Demographics/history	X	X			
Concomitant medication		X	X	X	X
Randomization	X	X			
Handing out of the decolonization set	X	X	X		
Questionnaire			X	X	
Assessment of compliance		X	X	X	X
Adverse events		X	X	X	X
Study end				X	X

X = Task fullfilled at this study time point

•Visit 1—Enrollment (Day 1).

•Visit 2—End-of-treatment (EOT) visit—day of surgery or days 1–2 after surgery.

•Visit 3—Test-of-cure (TOC) visit (clinical surgical control)—day 42 (± 14 days).

•Follow-up for implant-related or bone surgery after 1 year (± 2 months).

### Enrolment (visit 1)

The information collected during the routine pre-surgical consultation, and during orthopedic surgery, is not study-specific. This general data will be used as general demographic information and medical history within the study, in case of study participation. Moreover, we usually request consent for review of participants’ medical records and for the collection of blood and tissue samples to assess two intraoperative microbiological samples to diagnose possible infection. Likewise, we concomitantly might collect and storage biological specimens, or infection-controll-associated data and specimens, for genetic or molecular analysis in this trial and for future use in ancillary studies, which, however, is no study requirement in the BALGDEC trial. If a patient appears to be eligible, the following study-specific procedures are performed:

1. Patient information and obtaining written informed consent.

2. Assign a study identification number.

3. Record/complete medical history and demographics.

4. Review inclusion/exclusion criteria.

5. Randomize the patient and handout the decolonization set and the questionnaire.

### Visit 2 (end-of-treatment visit)

1. Record any additional interventions required.

2. Recuperate the empty packages and the questionnaires (only for decolonized patients).

3. Assess all adverse events of decolonization and related to the trial.

### Visit 3 (test-of-cure visit)

Every effort will be made to ensure that the final efficacy assessments (i.e., primary outcome data) are available for all study participants. Outpatients should return to the clinic (assessments can be also performed in the hospital), where the following assessments will be performed:

1. Assess all past adverse events of surgery, hospitalization, and decolonization.

2. Record all clinical and microbiological SSIs and its treatment and wound problems (if any).

For the study database, we will have assessed the following variables: patient’s characteristics (age, biological sex, body mass index, renal insufficiency, cirrhosis, other immune-suppressions, diabetes, pregnancy), indication of surgery, presence of osteosynthesis, all postoperative complications, SSI and pathogens, all adverse events during the study period, length of hospital stay, duration of eventual VAC (vacuum-assisted closure)/PICO use [19], and the patient’s opinion on the decolonization set (questionnaire) immediately after surgery. Table 3 indicates the timepoints of different assessments. These assessments are performed by experienced surgeons and study nurses but not by the infection control team that includes the patients to the trial. The outcome assessors are not blinded to the study arms. The future data analysts remain blinded during the interventional phase, but not during the final and interim data analysis, as they are also clinicians with full access to the individual patients’ electronic datafiles.

### Questionnaire

The previously validated questionnaire will be in German language with a total of seven predefined and open questions regarding the difficulties of decolonization, the completeness of scheduled actions, all adverse events during decolonization and surgery, and two questions regarding the scientific comprehension about the procedure (indication for decolonization, potential benefit expected in the individual case). The infection control nurses hands out the questionnaire at enrolment. If the patient has not filled it in until hospitalization, the infection control nurses will fill it in together with the patient, bedside and immediately after surgery.

### Follow-up for bone surgeries with implants in place

Usually, patients with implant-related orthopedic surgery return for a routine surgical control after 1 year. If this is not the case, the study nurse or the study investigators might phone the patient for a follow-up information regarding the study outcomes.

### Risks of the trial for participants

All patients can witness adverse events related to decolonization products or the surgical procedures. One theoretical risk could be a transient skin irritation, or allergy, to octenidine, which we assess in full detail. Overall, we expect no substantial adverse events according to reports from other centers and colleagues who already use the set for decolonization. Of note, the commercial set is in widespread use since 2016 and freely available on the Swiss market.

## Participant timetable

For this trial, we probably need 24 months, starting on February 27, 2023 (Table 4).

**Table 4:**
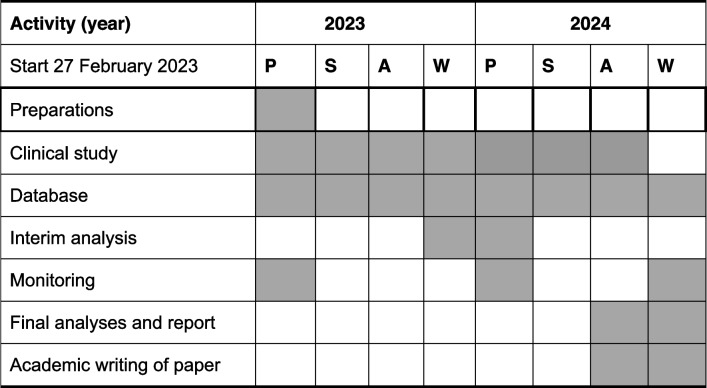
Time table of the BALGDEC trial

Time table: *P* Spring, *S* Summer, *A* Autumn, *W* Winter

## Monitoring and potential audits

The Unit for Clinical and Applied Research (UCAR) will assign an independent monitor with experience in prospective randomized trials. The monitor verifies all, or a part of the case report forms (CRF), data and written informed consents. According to the monitoring plan, the first visit will occur prior to the start, the second during the interim analyses, and the last visit at the study end (Table 5). A quality assurance audit/inspection may be conducted by the competent authority. The auditor/inspectors have access to all medical records, the investigator’s study files and correspondence, and the informed consent forms. The principal investigator and the sponsor will allow the persons being responsible for the audit to have access to the source data. All involved parties will keep the patient data strictly confidential.

**Table 5:** Monitoring plan

Study period	Time	Monitoring
Before study	January to February 2023	Monitoring will be informed about study conduct concerning data sampling and safety reporting Monitor controls if: • Documents are approved • Documents are at site • Investigators are familiar with study protocol and safety reporting • Investigators know their duties and responsibilities
During study	Spring 2024	All subjects: SDV for existence and informed consent First trial participant and at least 10% of trial participants recruited at the time of the monitoring visit, as far as available: eligibility, primary endpoint, SAEs
Study end	December 2024 to January 2025	Control for completeness of source data

## Statistical analyses, sample size calculations, and recruitment potential

### Main hypotheses

Among our selected study participants with an elevated risk for SSI and wound revisions, the pre-surgical decolonization may reduce the incidence of unplanned surgical revisions by 5% (from 10% without decolonization to 5% with decolonization).

### Determination of sample size

In our hospital, postoperative wound problems occur in at least 5% of all orthopedic interventions. However, the incidence for revision surgery in our particular study population is 10%, according to our clinical experience. We perform a superiority RCT with a power of 80% in favor of the decolonization. With 95% event-free surgeries in the decolonization arm versus 90% in the standard arm, we formally need 2 × 474 orthopedic surgery episodes, which we round up to 2 × 500 surgeries (*n* = 1000). For the secondary outcomes (adverse events, questionnaires), we have no formal, or minimal, sample sizes required.

### Planned statistical analyses

First, all analyses will be performed for the entire study population. In the second step, all analyses will be separately performed within substrata of patients, which are based on the type of orthopedic procedures (e.g., arthroplasties, implant-related surgeries) and the patients’ demographic parameters (elderly patients, immune suppression, high ASA scores). We will use descriptive statistics and compare groups using the Pearson *χ*^2^ test, the Fisher exact test, or the Wilcoxon rank-sum test, as appropriate. We will also recur to composite (SSI and wound problems) and separated (SSI; wound problems) multivariate analyses using a Cox regression model targeting the primary outcome variables. Variables with a *p*-value ≤ 0.2 in univariate analysis will be included in a stepwise forward selection process for multivariate analysis. Key variables will be checked for interaction. The number of variables in the final model is limited to the ratio of 1 outcome variable to 5 to 8 events [22]. The significance level is *p* ≤ 0.05 (two-tailed). We will use STATA™ (Version 15, College Station, TX, USA).

### Interim analysis and early termination

We will perform one interim analysis 1 year (± 2 months) after the inclusion of the first patient. If the result of group comparison between the decolonization and non-decolonization arms are statistically significant regarding the study objectives, the independent data monitoring committee will decide upon the interruption, or early termination, of the trial. Otherwise, the study continues. This committee will be composed of physicians and nurses with clinical experience in orthopedic infections and related research, who are not part of the Investigators of the BALGDEC trial. The data monitoring committee has also the right to call on a premature, additional interim analysis. We might also perform a futility analysis to check if the expected statistical power for the final analysis will not be < 30%. If it is lower than 30%, we will consider the trial will not be able to demonstrate the result, and the recruitment is no more ethical [23]. To balance (at least partially) for a potential power loss, we also may recruit 100 supplementary patients per arm, i.e., 600 episodes in each randomization arm.

### Final analyses

The intent-to-treat (ITT) population will consist of all randomized patients. Patient disposition and baseline characteristics will be based on the ITT population. The per-protocol (PP) population will consist of all patients who complete the study (or who are otherwise defined as a treatment failure) according to the clinical investigation plan and who have not deviated significantly from the protocol. All efficacy analyses will be repeated using the PP population.

### Handling of missing data and drop-outs

Significant missing data regarding the decolonization and outcomes will lead to a patient dropout. Drop-outs will be reported in the “Methods” section and excluded from both the ITT and the PP populations. However, due to the relatively short intervention period, we do not expect many missing data and renounce on imputations. The independent data monitoring committee may help in case of difficult interpretations of available data.

## Ethical and regulatory aspects

### Study registration

The study is registered in the Swiss Federal Complementary Database (BASEC 2023–00095) and in the international registry ClinicalTrials.gov (Number NCT05647252) in line with the requirements of the World Health Trial Registration Data Set. Supplementary file 1 is the original study protocol.

### Categorization of this study, safety reports and eventual amendments

This study makes use of a decolonization set that is already authorized in Switzerland. The indication and the dosage are used in accordance with the prescribing information. The study protocol will not be changed or amended without prior ethical committee’s approval. Premature interruption is reported within 30 days. The regular study end is reported to the ethical committee within 90 days, the final study report within 1 year. The ethical committee and authorities will receive annual safety reports. The study will be carried out in accordance with the Declaration of Helsinki, the guidelines of Good Clinical Practice, and the Swiss regulatory authority’s requirements.

### Patient information and early termination of the study

The investigators will inform potential participants about the study, its voluntary nature, procedures involved, expected duration, potential risks and benefits, and any potential discomfort. All participants will be provided an information sheet and informed consent form. The original form stays in the study records. The investigators uphold the principle of the participant’s right to privacy and that they shall comply with applicable privacy laws. Subject confidentiality will be further ensured by code numbers corresponding to the computer files. For verification, the ethics committee and regulatory authorities may require access to medical records, including the medical history. The sponsor may terminate the study prematurely in certain circumstances, e.g., ethical concerns, insufficient recruitment, safety issues, alterations in accepted clinical practice making the continuation unwise, or early evidence of benefit or harm of the experimental intervention. All patients are free to withdraw from participation in this study at any time, for any reason, and without prejudice. The withdrawal will not affect the actual medical assistance or future surgical treatments. On rare occasions, the investigators may terminate a patient’s participation to protect his/her best interest. After study termination, the evaluations required at the clinical visits will remain.

## Risk/benefits of the BALGDEC trial

All patients can witness adverse events (AE) related to surgical procedures and decolonization. A theoretical risk could be a higher incidence of SSI and related wound problems in the non-decolonization arm. Patients in the decolonization arm could witness more skin irritation and intolerance to octenidine and/or ingredients. Their potential benefits are a reduction of SSIs and wound problems in the decolonization arm. Supplementary file 2 is the “model consent form” in English language of the original form in German language.

## Safety

All orthopedic surgeries will be performed in the participation of experienced surgeons. The decolonization set is a commercial product in use since 2016. We expect no major adverse events of the product. An annual safety report is submitted once a year to the local ethics committee via the lead investigator. We, moreover, will perform interim (futility) analyses.

### Reporting and handling of pregnancies

The use of topical formulations containing octenidine is not a known danger for the fetus and the breastfed newborn [7, 8]. However, for purely formality reasons, we will exclude pregnant and/or breastfeeding women. Any pregnancy during the treatment phase of the study and within 30 days after discontinuation of study medication will be reported to the sponsor-investigator within 24 h. The course and outcome of the pregnancy will be followed up carefully, and any abnormal outcome regarding the mother or the child should be reported.

### Definition and assessment of (serious) adverse events and other safety related events

An adverse event (AE) is any medical occurrence in a study participant, which does not necessarily have a causal relationship with the study procedure. A serious adverse event (SAE) is classified as any untoward medical occurrence that results in death, is life-threatening, and results in hospitalization or a significant prolongation of hospitalization and persistent or significant disability. The investigators make a causality assessment. All SAEs are reported within 24 h to the sponsor-investigator. SAEs resulting in death are reported to the ethics committee within 7 days. The sponsor-investigator reports the safety signals within 7 days to the local ethics committee. Patients with AE and leaving the study will be treated off-study, without restriction, at the study site.

### Follow-up of (serious) adverse events

Participants terminating the study (either regularly or prematurely) with reported ongoing SAE, or any ongoing AEs of laboratory values or of vital signs being beyond the alert limit, will return for a follow-up investigation. This visit will take place up to 30 days after terminating the treatment period. Follow-up information on the outcome will be recorded on the respective AE page in the case report forms. Source data have to be available upon request. In case of participants are lost to follow-up, efforts will be made and documented to contact the participant to encourage him/her to continue study participation as scheduled. In case of minor AE, a telephone call to the participants is acceptable. All new SAE or pregnancies that the investigators will be notified of within 30 days after discontinuation of investigational product will be reported in appropriate report forms.

### Data handling and record keeping/archiving

Data is only saved, and stored, using the secured software REDCap®. Data can only be accessed by defined investigators. An electronic case report form is generated for every study participant. All data will be recorded by study nurses of the UCAR. The ID numbers are assigned by the REDCap® system. Corrections can only be made by authorized persons.

### Analysis and archiving

For data analysis, subject-related data from REDCap® will be exported and analyzed in a statistical software (STATA™). All health-related data will be archived in the REDCap®. Before data export, all patient identifiers are removed. All data will be stored for a minimum of 10 years. Collection, disclosure, and storage of data is carried out in accordance with Swiss data protection regulations and the Human Research Act.

## Discussion

Conceptually speaking, a preoperative decolonization over several days makes sense. The human body surface carriage of *S. aureus* [1, 16, 24] is an established risk factor not only for staphylococcal SSIs but also for community-acquired soft tissue infections [24] due to *S. aureus*. Indeed, in an epidemiological survey of 670 adult patients hospitalized for staphylococcal soft tissue infections in Geneva, Switzerland, 124 patients (12%) developed a new nosocomial or community-acquired soft tissue infections during their lifetime, mostly again due to *S. aureus*. Among the index cases with *S. aureus* infection, 92 (92/670; 14%) had another soft tissue infection, compared to 32 (32/353; 9%) non-staphylococcal index infections (Pearson *χ*^2^ test; *p* = 0.03). Equally, patients with initially *S. aureus* infections (compared to an index infection due to other bacteria) had a higher rate of another orthopedic infections due to *S. aureus* (70/86 vs. 16/86; *p* < 0.01). Of note, in that study, the time span between the patient’s first and last consultation (for any reason) was 21 years [24]. *S. aureus* can recolonize the skin and nares rapidly, even after long systemic antibiotic treatments.

Skin and mucosal carriage of *S. aureus* chronically occurs for 20–30% of all humans [24–26], and skin carriage still may persist in 15% of all patients despite use of sophisticated algorithms and decolonization protocols [23, 27]. Interestingly, this *S. aureus* carriage is not necessarily monoclonal. Different strains of *S. aureus* can coexist together [28] and together with other coagulase-negative staphylococci [11, 13]. Moreover, and epidemiologically speaking, a body colonization with methicillin-susceptible *S. aureus* (MSSA) does not protect from a nosocomial acquisition of MRSA carriage [28]. The literature provides several possible explications for the tenacity of *S. aureus* on the human skin and nasal mucosal surfaces, even in absence of foreign material [13]. The reasons are multi-factorial and extremely personalized [26]. Mechanisms to evade acquired and innate host defenses such as antimicrobial peptides [25] certainly play a role. Microbiota considerations [26], including gene products that protect against reactive oxygen and desiccation [25], are other fields of emerging science explaining this long-term carriage. *S. aureus*’ arsenal against elimination is further built up with modification of clumping factors, defensins, carbohydrate modifications, mannose-binding lectins, and other products [25]. Probably, the genetic background of the host plays an important role, too. Transient or persistent *S. aureus* colonization induces specific immune responses [25]. Humoral responses are the most studied, and little is known of cellular responses [26, 29]. However, even if human antibody response to *S. aureus* bacteremia differs between known chronic carriers and non-carriers, antibodies are not strong enough to prevent future infections [25]. An effective vaccine remains so far illusive [26, 29].

However, *S. aureus* is not an exclusive pathogen of orthopedic SSIs. Other skin commensals can equally provoke SSI or wound problems. Especially in implant-related infections, almost all skin commensals can cause infection. Topical skin antiseptics such as octenidine [7, 8] or polyhexanide [30] are ideal for the killing of the transient skin flora and the killing of a high proportion of coagulase-negative staphylococci in the deeper, sub-keratinous, flora. These are potent agents with less potential of developing resistances [31], or allergies, compared to mupirocin [32] or chlorhexidine [33], and can be easily applied for large body surfaces and mucosa [26, 29]. However, they are no absolute panacea either. Decolonization should be always applied within a bundle of other evidence-based measures [1, 13] and never alone.

The corresponding literature has been mainly published in the last two decades and was initially marked by many “before-and-after” reports in general surgery. If we consider the orthopedic literature separately, available data suggested that orthopedic patients may benefit of decolonization in a cost-saving way [1, 16, 34]. Wilcox et al. decreased the incidence of MRSA SSI from 2.3 to 0.33% after the introduction of intranasal mupirocin and triclosan showers before orthopedic surgery [35]. The same experience was repeated by others [36], sometimes also with nasal mupirocin use alone without concomitant body decolonization [37]. Kim et al. experienced that nasal mupirocin and chlorhexidine showers significantly reduced SSI risk among identified MRSA carriers hospitalized for elective orthopedic surgery [38]. Rao et al. reported that a preoperative decolonization protocol translated to an adjusted economic gain of US$ 230,000 to the facility [34]. In a multicenter before-and-after study, Wandhoff et al. investigated the efficacy of universal preoperative decolonization with polyhexanide in primary joint arthroplasty on SSIs [30]. Initial SSI rates due to *S. aureus* were 0.24/100 surgeries and decreased to 0.14/100 surgeries after introduction of decolonization [30]. Today, in many centers, the largest nosocomial pathogen group in orthopedic surgery are skin commensals other than *S. aureus* [11, 12, 15, 39]*.*

In contrast, there are also (recent) RCTs denying a beneficiary effect of decolonization in adult elective orthopedic surgery. For instance, a research group in Berne, Switzerland, which is a neighboring university hospital with a very similar infrastructure, successfully completed a prospective, randomized, single-blinded trial with 1318 adult patients [6]. The decolonization was 5 days of daily chlorhexidine showers and mupirocin nasal ointment twice a day. An interim analysis was performed after including half of the targeted *S. aureus* carriers (363 of 726). Based on the low infection rate in the control group (one of 179), a new sample size of 15,000 patients would have been needed. The authors found no difference in the risk of SSI between the decolonization and control groups, both in *S. aureus* carriers and noncarriers [6]. In January 2023, Lu et al. published a systematic review and meta-analysis regarding the association between nasal colonization of *S. aureus* and SSI in spinal surgery patients [40]. Although observational studies indicate that for example MRSA colonization increases the risk of SSIs in spinal surgery patients, an interventional nasal decolonization was unable to reduce the risk of overall SSIs in those carriers [40]. So far, various institutional recommendations emit different opinions. Depending on the level of evidence-based medicine required for recommendation, some suggest preoperative decolonization, while others do not.

### Strengths of the BALGDEC trial

Instead of implementing a logistically demanding measure in our service with more than 6000 annual surgical interventions, we investigate the decolonization among our high-risk patients for SSI. The main strength of the BALGDEC trial is the localization in a single-center, targeting only on orthopedic surgery performed by experienced orthopedic surgeons and the concentration on elderly and/or immune-compressed patients with high ASA scores. We regularly follow our patients postoperatively for several weeks, months, and years. It is very unlikely that these patients would be followed up only by the general practitioner or other orthopedic surgeons elsewhere in Switzerland. If there is no benefit of the cumbersome decolonization procedure with this target population, it is unlikely to be beneficiary for a larger patient population with lower ASA scores and lower ages. Moreover, our hospital has a long tradition to decolonize body MRSA carriage with octenidine. In that sense, the intervention is not new in terms of the choice of antiseptic agents. Further strengths are the randomized nature of the trial (in contrast to a before-after studies [28, 30, 35–38]), targeting all potential pathogens (not exclusively *S. aureus*), the improvement of the patient’s compliance by reducing the application period to only 5 days, the instruction of the patients by professional infection control nurses, and the evaluation of the decolonization procedure.

### Limitations

The limitations are the lack of control of the patient’s compliance, even if the trial allows decolonization by family members or nurses in elderly homes. Many patients are left for themselves to perform the decolonization at home. Unfortunately, we cannot hire personnel who supervises the decolonization, which would be very expensive. Likewise, for reasons of costs and the absence of clinical consequences, we renounce of the microbiological swabbing of healthy skin surfaces before and after the decolonization. Our study targets the clinical outcomes and not its microbiological surrogates. Lastly, the BALGDEC trial is not double-blinded and does not use placebo for various reasons. A true placebo-controlled decolonization is difficult to set up, in as much as the placebo agent must be entirely void of antiseptic effects and, at the same time, resemble to a body lotion. This is impossible. The daily act of decolonization, with any substance, is part of the preventive procedure. The decolonization is not only the application of an antiseptic substance but involves also daily showers and (mechanical) skin cleaning. Hence, also a placebo will still “decolonize.” Alternatively, we could test different decolonization products against each other, but this would be another study question. In this trial, we test a prevention concept, not substances.

## Conclusion

We are confident to detect a benefit of a presurgical decolonization of selected adult orthopedic patients using the commercial octenisan® set, in terms of the reduction of the SSI risk and associated wound problems. If our RCT confirms our hypothesis, future orthopedic patients with elevated risks of wound problems and SSI might benefit from this procedure.

### Supplementary Information

Supplementary Material 1: Supplementary file 1. Original protocol [41, 42].

Supplementary Material 2: Supplementary file 2. Model consent form.

Supplementary Material 3: Supplementary file 3. SPIRIT checklist [41, 42].

## Data Availability

We may provide anonymized key elements of the datasets upon reasonable scientific request.

## Abbreviations

AE: Adverse event
ASA: American Society of Anesthesiologists
ID: Infectious diseases
MRSA: Methicillin-resistant *Staphylococcus aureus*
RCT: Randomized controlled trial
SAE: Serious adverse event
SSI: Surgical site infection
UCAR: Unit of Clinical Applied Research (a professional unit entirely dedicated for clinical research in the Orthopedic Department at the Balgrist University Hospital in Zurich)
