# Supplementary material for: General skin and nasal decolonization with octenisan® set before and after elective orthopedic surgery in selected patients at elevated risk for revision surgery and surgical site infections—a single-center, unblinded, superiority, randomized controlled trial (BALGDEC trial)
Source: Trials. 2024 Jul 8;25:461. doi: 10.1186/s13063-024-08173-y (PMC11229206; doi:10.1186/s13063-024-08173-y)
Supplement: Supplementary file 1 — Supplementary Material 1: Supplementary file 1. Original protocol [41, 42]. [file 13063_2024_8173_MOESM1_ESM.pdf]

Clinical Investigation Plan

Study Type:	Clinical investigation with decolonization set
Study Categorisation:	Risk category A
Study Registration:	Clinicaltrials.gov und SNCTP
Sponsor-Investigator:	Prof. Dr. med. Mazda Farshad<br>Medical Director, surgeon-in-Chief, Orthopaedic Department<br>Balgrist University Hospital<br>Forchstrasse 340<br>8008 Zürich<br>044 386 1111<br>ilker.uckay@balgrist.ch
Principal Investigator:	Prof. Dr. med. Ilker Uçkay;<br>Head Infectiology, Head Clinical Research in Orthopaedic Department<br>Balgrist University Hospital<br>Forchstrasse 340<br>8008 Zürich<br>044 386 1111<br>ilker.uckay@balgrist.ch
Investigational Products:	octenisan® set (composed of octenisan® wash lotion and octenisan® md nasal gel)
Protocol Version and Date:	Version 1.0, 12.11.2022

CONFIDENTIAL

The information contained in this document is confidential and the property of the department of orthopaedics and infectiology of the Balgrist University Hospital. The information may not - in full or in part - be transmitted, reproduced, published, or disclosed to others than the applicable Competent Ethics Committee(s) and Regulatory Authority(ies) without prior written authorisation from Balgrist University Hospital, except to the extent necessary to obtain informed consent from those who will participate in the study.

SIGNATURE PAGE

Study Title	General Skin and Nasal Decolonization with octenisan® set before and after Elective Orthopedic Surgery in Selected Patients at Elevated Risk for Revision Surgery and Surgical Site Infections<br>– a Single-Center, Unblinded, Superiority, Randomized-Controlled Trial

The Sponsor-Investigator and trial statistician have approved the protocol version 1.0, 19.05.2022, and confirm hereby to conduct the study according to the protocol, current version of the World Medical Association Declaration of Helsinki, ICH-GCP guidelines or ISO 14155 norm if applicable and the local legally applicable requirements.

Sponsor-Investigators: Prof. Dr. med. Mazda Farshad0

Zürich, 3.11.2023

Place/Date Signatures

Principal Investigators:

I have read and understood this trial protocol and agree to conduct the trial as set out in this study protocol, the current version of the World Medical Association Declaration of Helsinki, ICH-GCP guidelines or ISO 14155 norm and the local legally applicable requirements.

Site: Balgrist University Hospital
Forchstrasse 340
8008 Zürich
Switzerland

Principal Investigators: Prof. Dr. med. Ilker Uçkay

Zürich, 3.1.2023

Place/Date Signatures

TABLE OF CONTENTS

SIGNATURE PAGE **2**
TABLE OF CONTENTS **3**
SYNOPSIS **4-11**
STUDY SUMMARY IN LOCAL LANGUAGE **11**
ABBREVIATIONS **11**
STUDY SCHEDULE **13**
1. STUDY ADMINISTRATIVE STRUCTURE **14**
1.1 Sponsor-Investigator 14
1.3 Statistician ("Biostatistician") 14
1.4 Laboratory 14
1.5 Monitoring institution 14
1.6 Data Safety Monitoring Committee 14
1.7 Any other relevant Committee, Person, Organisation, Institution 14
2. ETHICAL AND REGULATORY ASPECTS **14**
2.1 Study registration 14
2.2 Categorization of study 14
2.3 Competent Ethics Committee (CEC) 15
2.4 Competent Authorities (CA) 15
2.5 Ethical Conduct of the Study 15
2.6 Declaration of interest 15
2.7 Patient Information and Informed Consent 15
2.8 Participant privacy and confidentiality 16
2.9 Early termination of the study 16
2.10 Protocol amendments 16
3. BACKGROUND AND RATIONALE **16-17**
3.1 Background Rationale 16
3.2 Study definitions 17
3.3 Clinical Evidence to Date 17
3.4 Dose Rationale 18
3.5 Explanation for choice of comparator 17
3.6 Risks/Benefits of the study and of BioBanking 17
3.7 Justification of choice of study population 18
4. STUDY OBJECTIVES **18**
5. STUDY OUTCOMES **18**
6. STUDY DESIGN **18**
6.1 General study design and justification of design 18
6.1.3 Study duration 19
6.2 Methods of minimizing bias 19
6.2.1 Method of assignment to treatment/intervention (randomization, stratification) 19
6.2.2 Blinding procedures 19
6.3 Unblinding Procedures 19
7. STUDY POPULATION **20**
7.1 Eligibility criteria 20
7.2 Recruitment and screening 20

7.3 Assignment to study groups ... 20
7.4 Criteria for withdrawal / discontinuation of participants ... 20
8. STUDY INTERVENTION ... 21
8.1 Investigational Products (treatment / medical device) ... 21
8.1.1 Intervention treatment ... 21
8.1.2 Control Comparator ... 21
8.1.3 Packaging, Labelling and Supply (re-supply) ... 21
8.1.4 Storage Conditions ... 21
8.2 Administration of experimental and control interventions ... 21
8.2.1 Experimental Intervention ... 21
8.2.2 Control Intervention ... 21
8.3 Dose modifications ... 21
8.4 Compliance with study intervention ... 21
8.5 Data Collection and Follow-up for withdrawn participants ... 21
8.6 Trial specific preventive measures ... 22
8.7 Concomitant Interventions (treatments) ... 22
8.8 Study Drug Accountability ... 22
8.9 Return or Destruction of Study Drug ... 22
9. STUDY ASSESSMENTS ... 22
9.1 Assessment of primary outcome ... 22
9.1.2 Assessment of other outcomes of interest ... 22
9.1.3 Assessment of safety outcomes ... 22
9.1.4 Assessments in participants who prematurely stop the study ... 22
9.2 Procedures at each visit for both prospective-randomized studies ... 22
9.2.1 Screening/Pre-procedure assessment/Visit 1 ... 23
9.2.2 Visit 2, 3, 4, and End of Treatment ... 23
9.2.3 Visit 5 (Test of Cure) ... 23
9.2.4 Early Termination of Study Patients ... 23
10. SAFETY ... 23
10.1 Drug studies ... 23
10.1.1 Treatment by specialists at UKB ... 23
10.1.2 Definition and assessment of (serious) adverse events and other safety related events ... 24
10.1.3 Reporting of serious adverse events (SAE) and other safety related events ... 25
10.1.4 Follow up of (Serious) Adverse Events ... 26
11. STATISTICAL METHODS ... 26
11.1 Main hypotheses ... 26
11.2 Determination of Sample Size ... 26
11.3 Planned Analyses ... 27
11.3.1 Interim analyses and early termination ... 27
11.3.2 Final analyses ... 27
11.4 Handling of missing data and drop-outs ... 27
12. QUALITY ASSURANCE AND CONTROL ... 27
12.1 Data handling and record keeping / archiving ... 27
12.1.1 Case Report Forms ... 28
12.1.2 Specification of source documents ... 28

12.1.3 Record keeping / archiving 29
12.2 Data Management System, access and back-up 29
12.2.1 Analysis and archiving 29
12.3 Monitoring....29
12.4 Audits and Inspections 29
12.5 Confidentiality, Data Protection....30
12.6 Storage of biological material and related health data....30
13. PUBLICATION AND DISSEMINATION POLICY.... 30
14. FUNDING AND SUPPORT.... 30
15. INSURANCE.... 30
16. REFERENCES.... 31

Appendix 1 32
Appendix 2 32

**Sponsor-Investigator(s)**	Prof. Dr. med. Mazda Farshad, Medical Director, Balgrist University Hospital
**Principal Investi-gator(s)**	Prof. Dr. med. Ilker Uçkay, Head of Clinical Research UCAR in the Department of Orthopedic Surgery (Unit of Clinical and Applied Research), Head Infectiology and Infection Control, Balgrist University Hospi-tal, Zürich, Switzerland
**Research Team/ Co-Investigators and Authors**	Nadja Bragatto-Hess, ICN, Thorsten Studhalter, INC, Ines Unterfrauner, MD, Ilker Uçkay, MD, Mazda Farshad, MD, Team of UCAR<br><br>Authors of the ultimate scientific publication: Unterfrauner I, Bragatto-Hess N, Studhalter T, Farshad M, Uçkay I; eventually other authors
**Donation and Support**	Schülke & Mayr GmbH will kindly donate the investigational product and will partially financially support the clinical investigation via third party funding, but will be exempt of the academic part of the research. Schülke will equally share the clinical investigation plan with the study investigators
**Study Title**	General Skin and Nasal Decolonization with octenisan® set before and after Elective Orthopedic Surgery in Selected Patients at Elevated Risk for Revision Surgery and Surgical Site Infections<br>– a Single-Center, Unblinded, Superiority, Randomized-Controlled Trial
**Short Title / Study ID**	The **BALGDEC** Trial (**Balg**rist **Dec**olonization)
**Protocol Version, Date**	Version 1.0; 12 November 2022 (protocol composed and shared with schülke)
**Trial registration**	Swiss National Clinical Trials Portal (SNCTP) and the international registry ClinicalTrials.gov. Publication of the study protocol in the Journal "Trials".
**Study category and Rationale**	Category A clinical investigation: The decolonization set (octenisan® set, composed of octenisan® wash lotion and octenisan® md nasal gel) has already been placed on the market since 2016
**Clinical Phase**	Post-market clinical investigation

**Background and Rationale**	The general decolonization of the human body surface and, of adjacent pathogen reservoirs such as the nose, by industrial antiseptic agents, before elective surgery[1-3] is recommended by the World Health Organization (WHO)[4] for the risk reduction of superficial and deep surgical site infection (SSI) in many surgical disciplines. Such a decolonization is regarded as evidence-based in the majority of randomized and before-after trials since two decades, although negative studies also exist[5,6].<br><br>In the orthopedic field, this decolonization is likely to be more effective in elective patients with a proven body colonization of *Staphylococcus aureus*[2,3], in orthopedic patients with an inherent high risk for SSI[3], for which the majority of future SSI pathogens are skin commensals and *S. aureus*. These (eventual and future) pathogens of SSI are accessible to the topical agents[7] during the index surgery, if the patients are well instructed and know how to decolonize themselves[5,6].<br><br>In contrast, this pre-surgical decolonization is only a supplementary measure in the bundle of all combined efforts to prevent revision surgery for infection. Its individual power is limited for young, healthy individuals, for SSIs caused by pathogens from the internal body sites (intestinal, urinary, gynecologic regions), infections acquired on the surgical ward[1], and in settings with less specialized surgeries and a low volume of surgical experience[1]. Hence, in trials including all patients (or only those carrying *S. aureus*), the beneficial effect of decolonization can be diluted and provoke an additional costly and cumbersome organizational procedure for the majority of patients with low to moderate SSI risks. Moreover, many surgical site infections in orthopedic surgery, especially in implant-related surgery, are more due to coagulase-negative staphylococci (CoNS) than for *S. aureus*[9]. The hallmark of the CoNS group is *S. epidermidis* with approximatively 70% resistance (in Switzerland) to standard prophylactic antibiotic agents[9], As *S. epidermidis* is part of the normal human flora, typically the skin flora, and, only becomes pathogenic under certain conditions, a screening for this microorganism is not feasible in common practice. while the prophylaxis-resistant part of all orthopedic SSI pathogens is at 30-50%[10].<br><br>In this single-center, prospective, randomized, and controlled superiority trial, which is planned over a period of two years, we target on an orthopedic patient population with an elevated risk for revision surgery and SSI[1] (considering our recommendations in the section "inclusion/exclusion criteria)[11]; independently of a known body carriage for *S. aureus*. This study will be performed with an existing "set" manufactured by Schülke & Mayr GmbH[7,8]. The "set" was placed on the market in 2016. The distribution of octenisan® wash lotion and octenisan® nasal gel in the form of a set (octenisan® set) largely streamlines and facilitates the organizational application and compliance efforts of the study. Academically and clinically, we will gain insight in the performance of decolonization for a patient population at particular risk of postoperative complications, where every effort to prevent infection is of importance.

**Study Conduct**	The BALGDEC trial is a single-center trial, beginning in February 2023. Only elective surgery patients with a focus on high risk patients for revision and /or SSI according to prior studies will be included, whereas patients with an extraordinary high risk for SSI or impossibility of decolonization will be excluded. At the Balgrist site, this patient population includes: immune-suppressed patients of any type, tumor surgery patients, revision surgery, patients with ASA-Scores 3-4 points or with an age of ≥ 80 years; independently of the presence of an orthopedic implant. The operational study team is mainly composed by two infection control nurses, an infectious diseases physician, an orthopedic surgeon with experience in decolonization studies, and the UCAR-team (Unit for Clinical and Applied Research; study nurses).<br><br>The infectious control nurses and the infectiologist provide will provide pre-operatively specific instructions regarding the decolonization measure o the patients, and randomize them (1:1) between the presence or absence of the "set" used. The decolonization set will be physically handed over during the inclusion process. The randomization occurs electronically or by prefabricated cards by another study team member not involved in the patient's information process. During decolonization, patients are asked to change the bed linen and underwear every day and not to share towels, clothes and other textiles with their family or pets. They may use their habitual parfums, but must not use other topical antiseptic agents, body lotions or moisturizer during the decolonization period.<br><br>The duration of pre-surgical decolonization is planned to be five days. However, when this pre-surgical time period is too short, the decolonization may also start at least 3 days before surgery and be continued up to 2 days post-surgery. During the post-surgery application, the patient will be washed with water and octenisan® by their treating nurses. The patient will also return the empty/used set and answer to a short questionnaire during their hospital stay. The study team will recuperate the questionnaire during hospitalization. We renounce on routine microbiological assessments of skin colonization, but the hospital's infection control can demand it for other reasons regarding infection control. Hence, skin colonization data will only be available for some selected patients that are screened for body carriage of antibiotic-resistant pathogens, according to the infection control recommendations in Switzerland[12].<br><br>There will be a Monitoring at study start, the interim analysis and at the end.<br><br>The primary study outcomes are unplanned revision surgery for SSI and wound problems at 42 days for all surgeries; or 1 year in case of an implant-related surgery. Secondary objectives are other unplanned revision surgeries for non-SSI reasons, all adverse events, as well as patient's subjective impression of the decolonization.<br>The treatment period includes the following daily study visits:<br>• Visit 1 - Enrollment (Day 1)<br>• End-of-Treatment (EOT) -Visit is the Day of Surgery or Day 1-2 after surgery<br>• Test-of-Cure-Visit (TOC) (clinical surgical control) - Day 42 (+/- 14 days)<br>• Further follow-up (eventually by phone call) for implant-related surgery - 1 year (+/- 2 months)
**Objective(s)**	<u>Primary objective(s)</u><br>To reduce the incidence of SSI (and other unplanned postoperative wound revisions) in a highly selected patient population with an elevated risk for SSI.<br><u>Secondary objectives</u><br>To show the safety and all potential (any) adverse events observed during the decolonization period and during the hospitalization for surgery.
**Outcome(s)**	<u>Primary outcomes</u><br>- Remission (and inversely superficial or deep-space SSI and revision surgeries for non-infected wound problems; composite primary outcome) at 6 weeks (and/or a 1 year for surgeries with implant)<br><br><u>Secondary outcomes:</u><br>- Unplanned revision surgery for non-infection problems in same time period<br>- All adverse events during decolonization and hospitalization for surgery<br>- Subjective opinion on the decolonization (only for patients being decolonized)

**Study design**	Prospective-randomized, controlled, unblinded, superiority trial
**Inclusion / Exclusion criteria**	<u>Inclusion criteria</u>:<br>• Age ≥ 18 years<br>• Patients with an age ≥ 80 years (as considered particularly at risk for SSI)<br>• Elective orthopedic surgery at the Balgrist<br>• Chronic immune-suppression (diabetes mellitus, active cancer, cirrhosis CHILD C, renal dialysis, untreated HIV disease, medicamentous immune-suppression equivalent to prednisone ≥ 10 mg/day)<br>• Elective surgery in ischemic skin (e.g. major amputations)<br>• Elective surgery on non-diabetic and non-infected ulcerated skin<br>• Tumor (oncologic) orthopedic surgery<br>• ASA-Scores 3-4 points<br><br><u>Exclusion criteria</u>:<br>• Elective revision orthopedic surgery due to orthopedic infection within the last 12 months in the same area of surgery<br>• Emergency surgery (defined as planned surgery within the next 48 hours)<br>• Surgery on infected skin; or surgery under antibiotic treatment for any reason<br>• "Diabetic foot surgery" (distinct clinical entity; defined as below the ankle)<br>• Body mass index ≥ 35 kg/m^2 (anticipated difficulty of effective decolonization)<br>• Pregnancy (formality reasons)<br>• Intolerance or allergy to octenidin and/or ingredients in the octenisan® set<br>• Use of any other topical antiseptic agents other than octenisan® set (except for the duration of one day)<br>• Patient unable to understand; or under legal guardian for medical decisions<br>• Anticipated clinical follow-up of less than 6 weeks after surgery.<br>• ASA-Scores 1-2, and ASA-Score 5 (high risk of postoperative complications)<br>• Known skin colonization with antibiotic-multiresistant Gram-negative organisms defined by infection control protocols of Switzerland[12].
**Measurements and procedures**	We assess:<br>– Patient's characteristics (age, sex, body mass index, renal insufficiency, cirrhosis, other immune-suppressions, diabetes, pregnancy), indication of surgery, presence of osteosynthesis, all postoperative complications, surgical site infections and pathogens, all adverse events during hospitalization, length of hospital stay, duration of an eventual VAC (vacuum-assisted closure) / PICO use[13], and the patient's opinion immediately (patient questionnaire) after surgery.<br>– The previously validated questionnaire will be in German language with five open questions: Practical difficulties of decolonization, the completeness of scheduled decolonization actions, all adverse events during decolonization and surgery, and two questions regarding the comprehension of the science (indication for decolonization, potential benefit expected in the individual case).
**Study Product**	Schülke & Mayr GmbH will provide 550 set for free of use for the BALGDEC trial.<br>We will use the prefabricated kits by Schülke & Mayr GmbH[7,8] with patient's information leaflets available in German, English, French and Italian languages.<br>The active ingredient contained in both products (octenisan® wash lotion and octenisan® md nasal gel, both products combined in octenisan® set) [8] (no. article 11636528, EAN 4032651979264) is octenidine dihydrochloride. One kit would cost on the Swiss market approximatively 22,25 CHF.-[8]<br>The wash lotion in the is applied once a day. The nasal gel is applied 2-3 times per day.[7,8]<br>The questionnaire is handmade and validated by the Investigators.

Timetable

P = Spring
S = Summer
A = Autumn
W = Winter

**Activity (year)**	**2023**				**2024**			
Start schedule February 2023	**P**	**S**	**A**	**W**	**P**	**S**	**A**	**W**
Preparations	■							
Clinical study	■	■	■	■	■	■	■	
Database	■	■	■	■	■	■	■	■
Interim analysis				■	■			
Monitoring	■				■			■
Final analyses and report							■	■
Academic writing of paper							■	■

Number of Participants with Rationale

Postoperative wound problems occur in at least 5% percent of all interventions. Incidence for both, revision surgery and/or SSI, in our selected patient population is set for 10%. We perform a superiority RCT with a power of 80% in favor of the decolonization. With event-free surgeries to 95% in the decolonization arm versus 90% in the standard arm, we formally need 2 x 474 surgery episodes, which we sum up to 2 x 500 surgeries (n = 1'000). A patient can be included several times as long as he/she gets not infected.

At the Balgrist, we perform at least 5000 surgical operations per year (in reality more than 6000). With a very conservative estimation, we see at least 600 surgical episodes per year (12% of surgeries) that can be included; mostly because of the advanced age, diabetes, and high ASA-Scores of our patients. The interventional part of the trial lasts two years; with interim analysis after 1 year (+/- 2 months). An independent advisory board might stop the trial. For the secondary outcomes (adverse events, questionnaire), we do not require a minimal sample size.

GCP Statement

This study will be conducted in compliance with the protocol, the Declaration of Helsinki, the ICH-GCP and national legal and regulatory requirements.

Statistical analyses

Group comparisons by Pearson-Chi^2-test or the Wilcoxon-ranksum-tests. Multivariate adjustments by Cox regression without cluster analysis. Stratifications according to the type of surgery. We will perform Intention-to-Treat and Per-Protocol analyses.

Budget

**Costs** *(cost-covering)*	Total	1st Year	2nd Year
a. Ethical Comittee	3,000	3,000	0
b. Monitoring	5,000	2,500	2,500
c. Nurse 20% for 2 years	39,000	19,500	19,500
d. Social securities	5,654	2,827	2,827
e. Miscellaneous	2,346	423	1,923
**Swiss Francs (CHF)**	**55,000**	**28,250**	**26,750**

Payment of donation

Installments in February 2023 to Balgrist funding account:

- Swiss Francs (CHF) 47,500 – Schülke Switzerland
- Installments in 2024 to third party funding account (End of Trial):
  CHF 7.500 – Schülke Switzerland

The 550 sets (500 for the study, 50 in reserve) are provided by Schülke Switzerland

Insurance

The Trial is covered by the research insurance of the Balgrist.

Monitoring

The Sponsor Investigator will assign and pay an independent Monitor. The Monitoring is scheduled for three times: begin of the trials, after one year and concomitant to the interim analyses, and at the study end.

**Storage of data and of the used kits**	All health-related patient data will be stored for at least 10 years; and archived in the data capture software REDCap™. Hence, the Case Report Form is in electronic form and electronically archived in RedCap. Patient-source data will be registered using subject identifiers. After full data analysis, all subject identifiers will be erased.<br>The decolonization kits will be taken from the patients after use; and counted. After the study, they will be destroyed.
**Jurisdiction**	The place of jurisdiction is the Canton Zürich, Switzerland.<br>The Donator and the Institution of the Sponsor will make a separate agreement document concerning jurisdiction issues.
**Publications**	The study team at the Balgrist will present the at least part of the interim results in scientific congresses; and will publish the final results in one or two scientific surgical journals. The Protocol will be published separately, e.g. Journal "Trials".
**Donation**	Schülke & Mayr GmbH will support the study with an unconditional donation of 55,000.- (fifty-five thousand) Swiss Francs (paid in two tranches), which will be used for the salary of the study / infection control nurses, the monitoring and for the Ethical Committee fees. The study team will not make financial profit and cover the minimal operational costs.<br>Schülke & Mayr GmbH will donate all the decolonization kits used for the study, for free (including the corresponding transport costs).<br>Schülke & Mayr GmbH has the rights to consult the data and the draft, but will not be part of the study / academic team that will publish the results of the BALGDEC study.

STUDY SUMMARY IN LOCAL LANGUAGE

Die Haut- und Nasendekolonisation von Patienten vor allgemeiner elektiver Chirurgie ist in mehreren Guidelines empfohlen. Die tägliche prä-operative Dekolonisation mit topischen Mitteln und Salben zielt auf die Reduktion von postoperativen Wundinfekten. Die Literatur unterstützt diese Maßnahme vor allem bei Patienten mit nachgewiesener Hautkolonisation mit *Staphylococcus aureus* und bei chirurgischen Eingriffen mit hohem Risiko von postoperativen Infekten. Der optimale Ablauf ist unbekannt. In der Literatur dauert sie zwischen drei bis sieben Tagen vor elektiver Chirurgie und betrifft meistens die alleinige Nasendekolonisation; und seltener die Ganzkörperdekolonisation mit einschließlich er Nasensalbe.

Auf der anderen Seite ist diese generelle Dekolonisation organisational sehr aufwändig und auch nicht immer erfolgreich in allen Studien, welche alle Patienten zusammen untersucht hatten. In der orthopädischen Chirurgie, in der die Infektionsrate traditionell niedriger ist als bei anderen chirurgischen Disziplinen, könnten eventuell nur ausgesuchte Patienten von der Dekolonisation profitieren. Zugleich sind orthopädische Operationen meistens mit Implantaten vergesellschaftet, dessen Infektionen mehrheitlich durch (Koagulase-negative) Hautkeime verursacht sind, welche wiederum (im Gegensatz zu *S. aureus*) nicht durch die perioperative Standard-Prophylaxe abgedeckt wären. Letztendlich spielt die Patientencompliance eine Rolle. Das Vorhandensein von Dekolonisations-Set wäre eine substantielle Vereinfachung der Prozedur, anstatt den Patienten ein Rezept abzugeben (welches diese verwenden oder nicht).

Gezielte prospektive-randomisierte Studien unter orthopädischen Patienten mit erhöhtem Infektrisiko, welche das Resultat der Dekolonisation von allen potentiellen (Prophylaxe-resistenten) Hautkeimen untersuchen sowie die Patienten-Compliance erfragen, existieren in der Literatur nur ansatzweise.

Wir möchten solch eine Studie durchführen mit Hilfe eine Dekolonisations-Sets. Diese Studie ist mit Kosten verbunden, welche freundlicherweise von der Firma Schülke mit einer Spende über 55‘000 CHF (20% Lohn einer Study Nurse während 2 Jahren) sowie einer Spende von 550 Dekolonisations-Sets unterstützt werden. Der Sponsor und er Principal Investigator sind vom Balgrist. Die Spende hat keinen Einfluss auf Durchführung und Publikation der Studie.

ABBREVIATIONS

AE	Adverse Event
ASA	American Society of Anesthesiologists
ASR	Annual Safety Report
CA	Competent authority
CEC	Competent Ethics Committee
CRP	Serum C-reactive protein
eCRF	Electronic case report forms
EOT	End of treatment
GCP	Good Clinical Practice
ICH-GCP	International Conference on Harmonization of Good Clinical Practice
IMM	Institut für Medizinische Mikrobiologie
NRS	Nutritional Risk Screening
REDCap	Research Electronic Data Capture
SAE	Serious Adverse Events
SNCTP	Swiss National Clinical Trials Portal
SSI	Surgical Site Infection
SOP	Standard Operation Procedure
SUSARs	Suspected Unexpected Serious Adverse Reactions
UCAR	Unit for Clinical and Applied Research
VAC	vacuum-assisted negative pressure
ZLZ	Zentrallabor Zürich

STUDY SCHEDULE

Randomized-controlled trial

**Study Periods**	**Screening/ Baseline***	**Visit 1*<br>Enrol-ment**	**Visit 2*<br>End-of-Treatment**	**Visit 3<br>Test-of-Cure**	**Follow-up for surger-ies with implants**
**Time**	Day -30 to 0	Enrolment	Surgery day or 1-2 days after the surgery	6 weeks<br>(+/- 14 days)	1 year<br>(+/- 2 months)
In-/ Exclusion criteria	X	X			
Informed consent	X	X			
Demographics / history	X	X			
Concomitant medication		X	X	X	X
Randomization	X	X			
Handing out of the decolonization set		X	X		
Questionnaire			X	X	
Compliance			X	X	X
Adverse Events		X	X	X	X
Study End				X	X

We assess:

Patient's characteristics (age, sex, body mass index, renal insufficiency, cirrhosis, other immune-suppressions, diabetes, pregnancy), indication of surgery, presence of osteosynthesis, all postopera-tive complications, surgical site infections and pathogens, all adverse events during hospitalization, length of hospital stay, duration of an eventual VAC (vacuum-assisted closure) / PICO use13, and the patient's opinion on the use of the decolonization set (patient questionnaire) immediately after surgery.

The previously validated questionnaire will be in German language with five open questions: Practical difficulties of decolonization, the completeness of scheduled decolonization actions, all adverse events during decolonization and surgery, and two questions regarding the comprehension of the science (indication for decolonization, potential benefit expected in the individual case).

1. STUDY ADMINISTRATIVE STRUCTURE

1.1 Sponsor-Investigator

Prof Dr. med. Mazda Farshad, Medical Director, Balgrist University Hospital, Forchstrasse 340, 8008 Zürich

The sponsor is responsible for trial design and management, data handling and record keeping, subject protection, quality management, financing, investigational product management and safety evaluation. He ensures oversight and designates appropriately qualified personnel. The sponsor is going to supervise data collection, management and integrity as well as analysis and interpretation.

1.2 Principal Investigator (PI)

Prof Dr. med. Ilker Uçkay, Head Infectiology, Head UCAR (Clinical Research in Orthopedic Department).

The PI is responsible for the protocol and GCP-conform conduct of the trial at the site. The PI delegates and supervises trial-related duties to qualified staff and ensures medical care of trial subjects. The PI ensures that randomization and informed consent procedures are followed, source and CRF records are accurate and that safety reporting requirements are met.

1.3 Statistician ("Biostatistician")

Statistical analyses will be performed by the investigators (and eventually the biostatistician Mr. Tobias Götschi of UCAR (Unit for Clinical and Applied Research) using SPSS™ and/or STATA™ software (Version 14). In case of necessity, other biostatisticians will be consulted.

1.4 Laboratory

Laboratory analysis will be done by ZLZ Zentrallabor Zürich and IMM, in the immediate vicinity of UKB, as part of the regular analysis of the clinical course.

1.5 Monitoring institution

An internal study monitoring board is established to perform ongoing study surveillance and to perform interim analyses if appropriate. UCAR (Unit for Clinical and Applied Research); Prof Dr. med. Ilker Uçkay, Balgrist Campus, Lengghalde 5, 8008 Zürich

1.6 Data Safety Monitoring Committee

A data safety committee of two persons with experience in clinical research and biostatistics who are not part of the investigators or future authors of the scientific publication will monitor the safety of data and of the study; during the interim analyses (approximatively after one and two years into the study).

1.7 Any other relevant Committee, Person, Organisation, Institution

The study takes place at the Balgrist University Hospital.

2. ETHICAL AND REGULATORY ASPECTS

The decision of the Competent Ethics Committee (CEC) concerning the conduct of the study will be made in writing to the Sponsor-Investigator before commencement of this study. The clinical study can only begin once approval from the CEC has been received. Any requirements imposed by the authorities shall be implemented.

2.1 Study registration

The study will be registered at http://www.clinicaltrials.gov and http://www.snctp.ch.

2.2 Categorization of study

Category A. The investigational products used in this study are already authorized in Switzerland for the prevention of surgical sites infections. The indication and the dosage are used in accordance with the product information and international guidelines. There will be no placebo.

2.3 Competent Ethics Committee (CEC)

The principal investigator ensures that approval from an appropriately constituted Competent Ethics Committee (CEC) is sought for this clinical study.

The reporting duties such as all changes in research activity, all unanticipated problems involving risks to humans and planned or premature study end and the allowed time frame are respected by this study. The study protocol will not be changed without prior Sponsor and CEC approval, except when it's necessary to eliminate apparent immediate hazards to study participants.

Premature study end or interruption of the study is reported within 15 days. The regular end of the study is reported to the CEC within 90 days, the final study report shall be submitted within one year after study end. Amendments are reported according to chapter 2.10.

2.4 Competent Authorities (CA)

CA (*swissmedic*) approval is only necessary for category B and C studies. Category A studies do not require CA approval. The CA is entitled to carry out inspections of all clinical trials.

2.5 Ethical Conduct of the Study

The study will be carried out in accordance to the protocol and with principles enunciated in the current version of the Declaration of Helsinki, the guidelines of Good Clinical Practice (GCP) issued by ICH, the Swiss Law and Swiss regulatory authority's requirements. The CEC and regulatory authorities will receive annual safety and interim reports and will be informed about study stop/end in agreement with local requirements.

2.6 Declaration of interest

No conflict of interest compromises the professional judgement of our investigators and the other involved people in conducting and reviewing this study. Their objectivity is not influenced in any way (e.g. independence, intellectual, financial, proprietary) or through any party.

However, Schülke GmbH supports the Trial with an unconditional grant (third party funding) to pay a minor part of the salary of the study nurse(s) and will provide the decolonization sets free of charge and for exclusive use in the study. Schülke GmbH will not have access to the individual non - anonymized data and will not interfere with the scientific publication of the final study results according to the terms specified in the contract of the clinical investigation .

2.7 Patient Information and Informed Consent

For the participation in the study, patients will be recruited/preselected by any of the investigators of the study. If patients match the inclusion criteria and do not meet any exclusion criterion, they will be informed by one of the study investigators, about the study, its nature, purpose, procedures involved, expected duration, participating investigators, potential risks and benefits and any potential discomfort the study could entail, during post-surgery visit when an orthopedic infection is diagnosed. Each participant will be informed that the participation in the study is completely voluntary and that he/she may withdraw from the study at any time and that withdrawal of consent will not affect his/her medical assistance and treatment in the future. No further screening requirements (other than the in- and exclusion criteria) exist.

All participants will be provided with a participant information sheet and informed consent form describing the study and entailing sufficient information for the participant to make an informed decision about their willingness to participate in the study. The patient information sheet and the consent form will be submitted to the CEC to be reviewed and approved. The information sheet provides the possibility to read through the study concept again and enables the patient to rethink the study participation without being pressured into deciding. If the participant decides to take part in the study, he/she will be asked to date and sign the informed consent form. The potential participant will be requested to read through the consent form and the information sheet carefully and to clarify any misunderstandings before signing. Once the patient dates and sign the informed consent form, one of the investigators will also date and sign the aforementioned document. The participant will be given a copy of the signed document. The original signed informed consent form will be retained as part of the study records.

The formal consent of a participant, using the approved consent form, must be obtained before the

participant is submitted to any study specific procedures.

The collection of the general consents for further use of health-related personal data and biological material is a standard at UKB. It is not study-specific.

2.8 Participant privacy and confidentiality

The investigators affirm and uphold the principle of the participant's right to privacy and that they shall comply with applicable privacy laws. Especially, anonymity of the participants shall be guaranteed when presenting the data at scientific meetings or publishing them in scientific journals.

Individual subject medical information obtained as a result of this study is considered confidential and disclosure to third parties is prohibited. Subject confidentiality will be further ensured by utilising subject identification code numbers to correspond to treatment data in the computer files.

For data verification purposes, authorised representatives of the Sponsor (-Investigator), a competent authority (e.g. *Swissmedic*), or an ethics committee may require direct access to parts of the medical records relevant to the study, including participants' medical history.

2.9 Early termination of the study

The Sponsor-Investigators may terminate the study prematurely according to certain circumstances, for example:

- ethical concerns,
- insufficient participant recruitment,
- when the safety of the participants is doubtful or at risk, respectively,
- alterations in accepted clinical practice that make the continuation of a clinical trial unwise,
- early evidence of benefit or harm of the experimental intervention, e.g. based on interim analyses

2.10 Protocol amendments

Substantial amendments are only implemented after approval of the CEC.

Under emergency circumstances, deviations from the protocol to protect the rights, safety and well-being of human subjects may proceed without prior approval of the CEC. Such deviations shall be documented and reported to the CEC as soon as possible.

All non-substantial amendments are communicated to the CEC within the Annual Safety Report (ASR).

As substantial Amendments Count:

a) Changes, which affect security and health of the participants or their rights and duties.
b) Changes of the study protocol, due to new scientific findings, which affect study arrangement, study methods, objectives or statistical analysis.
c) Changes of the study location or inclusion of an additional study location.
d) Personnel changes, such as change of the sponsor-investigator.

3. BACKGROUND AND RATIONALE

3.1 Background Rationale

The general decolonization of the human body surface and, of adjacent pathogen reservoirs such as the nose, by industrial antiseptic agents, before elective surgery[1-3] is recommended by the World Health Organization (WHO)[4] for the risk reduction of superficial and deep surgical site infection (SSI) in many surgical disciplines. Such a decolonization is regarded as evidence-based in the majority of randomized and before-after trials since two decades, although negative studies also exist[5,6].

In the orthopedic field, this decolonization is likely to be more effective in elective patients with a proven body colonization of *Staphylococcus aureus*[2,3], in orthopedic patients with an inherent high risk for SSI[3], for which the majority of future SSI pathogens are skin commensals and *S. aureus*. These (eventual and future) pathogens of SSI are accessible to the topical agents[7] during the index surgery, if the patients are well instructed and know how to decolonize themselves[5,6].

In contrast, this pre-surgical decolonization is only a supplementary measure in the bundle of all combined efforts to prevent revision surgery for infection. Its individual power is limited for young, healthy individuals, for SSIs caused by pathogens from the internal body sites (intestinal, urinary, gynecologic regions), infections acquired on the surgical ward[1], and in settings with less specialized surgeries and a low volume of surgical experience[1]. Hence, in trials including all patients (or only those carrying *S. aureus*), the beneficial effect of decolonization can be diluted and provoke an additional costly and cumbersome organizational procedure for the majority of patients with low to moderate SSI risks. Moreover, many surgical site infections in orthopedic surgery, especially in implant-related surgery, are more due to coagulase-negative staphylococci (CoNS) than for *S. aureus*[9]. The hallmark of the CoNS group is *S. epidermidis* with approximatively 70% resistance (in Switzerland) to standard prophylactic antibiotic agents[9], As *S. epidermidis* is part of the normal human flora, typically the skin flora, and, only becomes pathogenic under certain conditions, a screening for this microorganism is not feasible in common practice. while the prophylaxis-resistant part of all orthopedic SSI pathogens is at 30-50%[10].

In this single-center, prospective, randomized, and controlled superiority trial, which is planned over a period of two years, we target on an orthopedic patient population with an elevated risk for revision surgery and SSI[1] (considering our recommendations in the section "inclusion/exclusion criteria)[11]; independently of a known body carriage for *S. aureus*. Academically and clinically, we will gain insight in the performance of decolonization for a patient population at particular risk of postoperative complications, where every effort to prevent infection is of importance.

3.2 Investigational Product and Indication

This study will be performed with an existing "set" manufactured by Schülke & Mayr GmbH[7,8]. The "set" was placed on the market in 2016. The distribution of octenisan® Set largely streamlines and facilitates the organizational application and compliance efforts of the study. The active substance is octenidine dihydrochloride (Appendix 1). The questionnaire is designed and validated by the Investigators at the Balgrist (Appendix 2).

3.2.1 Study definitions

An orthopedic infection for this trial is the microbiological evidence of bacteria in at least two intraoperative tissue samples together with radiological (osteomyelitis, collections, inflammation) and/or clinical evidence of infection (pus, discharge, sinus tracts, rubor, calor, pain). Histological proof is facultative for this study. Implants are defined as any implants except for transient wires or fixator pins. A wound problem is any unplanned post-surgical wound pathology leading to surgical revision. Remission of infection is the absence of clinical and/or radiological and/or laboratory signs of (former) infection after the minimal follow-up time of 1 year for implant -related surgery with implants left in place; or two months for implant-free surgery (including surgery when all implants have been removed)..

3.3 Clinical Evidence to Date

See chapter 3.1

3.4 Dose Rationale

n.a; the investigational products will be applied as per product information.

3.5 Explanation for choice of comparator

See chapter 3.1

3.6 Risks/Benefits of the study

All patients can witness adverse events related to surgical procedures and decolonization, which however, are related to the therapy itself, and not to the specific study protocol. A theoretical risk could be a higher incidence of SSI and related wound problems in the Non-Decolonization arm. Patients in the decolonization arm could witness more skin irritation, intolerance and allergy to octenidin and/or ingredients of the formulations. The potential benefits are a reduction of the SSI risk and related wound problems in the decolonization arm.

Pregnancy and breast-feeding

The use of topical formulations containing octenidin is not a known danger for the fetus and the breast-fed newborn[7,8]. However, due to formality reasons inherent to most interventional trials, we exclude pregnant or breastfeeding women from the trial.

3.7 Justification of choice of study population

See chapter 3.1. No vulnerable participants are included.

4. STUDY OBJECTIVES

To reduce the incidence of SSI (and other unplanned postoperative wound revisions) in a highly selected patient population with an elevated risk for SSI. To show the safety and all potential (any) adverse events observed during the decolonization period and during the hospitalization for surgery.

5. STUDY OUTCOMES

Primary outcomes:

- Remission (and inversely superficial or deep-space SSI and revision surgeries for non-infected wound problems; composite primary outcome) at 6 weeks (and/or a 1 year for surgeries with implant)

Secondary outcomes:

- Unplanned revision surgery for non-infection problems in same time period
- All adverse events during decolonization and hospitalization for surgery
- Subjective opinion on the decolonization (only for patients being decolonized)

6. STUDY DESIGN

6.1 General study design and justification of design

This is a prospective-randomized, controlled, unblinded, superiority trial, completed with a questionnaire of 5 key questions regarding the decolonization. We assess the following variables:

6.1.2 In case of refusal or withdrawal from the study

When a patient refuses to take part in the study, or is rejected by the investigators, his/her treatment will continue according to usual therapeutic standards and follow-ups. If a patient's withdrawals his/her consent during the study period or up to one month after the last study visit, his/her information and results will be deleted from further analyses.

6.1.3 Study duration

For the interventional part of the study, we need 24 months; starting in February 2023 (CEC approval provided). The following Table highlights some key time events scheduled for the current study.

Timetable

P = Spring
S = Summer
A = Autumn
W = Winter

**Activity (year)**	**2023**				**2024**			
Start schedule February 2023	**P**	**S**	**A**	**W**	**P**	**S**	**A**	**W**
Preparations	■							
Clinical study	■	■	■	■	■	■	■	
Database	■	■	■	■	■	■	■	■
Interim analysis				■	■			
Monitoring	■				■			■
Final analyses and report							■	■
Academic writing of paper							■	■

6.2 Methods of minimizing bias

The methods of minimising bias applied in our study are the randomization to add validity of the statistical tests used to demonstrate significance. The differences between intervention and control groups should behave like differences between two random samples from the population so that they can be compared to what would be expected in the population by chance.

6.2.1 Method of assignment to treatment/intervention (randomization, stratification)

After written informed consent given participants will be randomized with a 1:1 ratio in either treatment group. Randomization is done by designated study staff drawing a sealed envelope containing a randomization card or electronically. Patients are informed about the assignment by the investigators.

6.2.2 Blinding procedures

There will be no blinding of patients, and no placebos.

6.3 Unblinding Procedures

n.a.

7. STUDY POPULATION

7.1 Eligibility criteria

Inclusion criteria:

- Age ≥ 18 years
- Patients with an age ≥ 80 years (as considered particularly at risk for SSI)
- Elective orthopedic surgery at the Balgrist
- Chronic immune-suppression (diabetes mellitus, active cancer, cirrhosis CHILD C, renal dialysis, untreated HIV disease, medicamentous immune-suppression equivalent to prednisone ≥ 10 mg/day)
- Elective surgery in ischemic skin (e.g. major amputations)
- Elective surgery on non-diabetic and non-infected ulcerated skin
- Tumor (oncologic) orthopedic surgery
- ASA-Scores 3-4 points

Exclusion criteria:

- Elective revision orthopedic surgery due to orthopedic infection within the last 12 months in the same area of surgery
- Emergency surgery (defined as planned surgery within the next 48 hours)
- Surgery on infected skin; or surgery under antibiotic treatment for any reason
- “Diabetic foot surgery” (distinct clinical entity; defined as below the ankle)
- Body mass index ≥ 35 kg/m^2 (anticipated difficulty of effective decolonization)
- Pregnancy (formality reasons)
- Intolerance or allergy to octenidin and/or ingredients in the octenisan® set
- Use of any other topical antiseptic agents other than octenisan® set (except for the duration of one day)
- Patient unable to understand; or under legal guardian for medical decisions
- Anticipated clinical follow-up of less than 6 weeks after surgery.
- ASA-Scores 1-2, and ASA-Score 5 (high risk of postoperative complications)
- Known skin colonization with antibiotic-multiresistant Gram-negative organisms defined by infection control protocols of Switzerland[12].

7.2 Recruitment, screening and patient’s instructions

The concerning patients will be screened every day by the clinicians and the study investigators during the presurgical surgical and anesthesiologic consultations. In case all inclusion and no exclusion criteria are met, the patient will be informed about the study by one of the investigators (see chapter 2.7) and orally instructed about he handling of the decolonization set. It is important that the study investigator instructs the patient (and his(her accompanying family members) directly. An instruction of the set handling by the surgeons or anesthesiologists alone will not be accepted.

7.3 Assignment to study groups

Assignment to the study group (control versus intervention group) will be done with randomization cards (or electronically).

7.4 Criteria for withdrawal / discontinuation of participants

All patients are free to withdraw from participation in this study at any time, for any reason, and without

prejudice. A patient who withdraws consent by refusing to continue with study procedures/observations will be terminated from the study. The reason for withdrawal of consent will be clearly documented wherever possible. However, it is not required for patients to provide their reason.

The investigator should make every effort to address non-compliance issues and ensure that relevant study data are obtained from patients whenever possible.

To enable collection of follow-up data, the investigator may stop study treatment at any time without withdrawing the patient from the study (e.g. the patient experiences intolerable or unacceptable AEs possibly related to study treatment and where such treatment cannot be modified within the confines of the protocol).

On rare occasions, the investigator may exclude a patient from the study to protect his/her best interest e.g. to protect them from excessive risk or risk with a demonstrated lack of benefits (serious side-effects) or to maintain the integrity of the data (when participates are not following study procedures or may be deliberately providing false information). The investigator must explain to the participant the reasons. If a patient is withdrawn before completing the study, the reason for withdrawal will be entered in the electronic case report form (eCRF). Whenever possible and reasonable, the evaluations that are required at the next scheduled visit will be performed at early termination.

8. STUDY INTERVENTION

8.1 Investigational Products (treatment / medical device)

Schülke & Mayr GmbH will provide 550 octenisan® sets free of charge for use during the BALGDEC trial. The sets will be used according to the instruction provided in the patient information leaflet. The instructions will be available in German, English, French and Italian languages.

The active ingredient contained in octenisan® set (octenisan® wash lotion and octenisan® md nasal gel, [8] no. article 11636528, EAN 4032651979264) is octenidine dihydrochloride. One set costs on the Swiss market approximatively 22,25 CHF.-[8]

The wash lotion is applied once a day. The nasal gel is applied 2-3 times per day.[7,8]

8.1.1 Intervention treatment

The duration of pre-surgical decolonization is planned to be five days. However, when this pre-surgical time period is too short, the decolonization may also start at least 3 days before surgery and may be continued up to 2 days post-surgery. In case of post-surgery application, the patient will be washed with water and octenisan® wash lotion by their treating nurses. The patient will also return the empty/used set and answer to a short questionnaire during their hospital stay. The study team will recuperate the questionnaire during hospitalization.

During decolonization, patients are asked to change the bed linen and underwear every day and not to share towels, clothes and other textiles with their family or pets. They may use their habitual perfumes, but must not use other antiseptic agents, body lotions or moisturizer during the decolonization period.

8.1.2 Control Comparator

Absence of decolonization and absence of the questionnaire.

8.1.3 Storage Conditions

The sets can be stored at ambient temperature for several months, as indicated on the packaging of both investigational products. They will be in the (locked) office of the Infection Control nurses and in the Prof. Uçkay's office.

8.2 Dose modifications

n.a.

8.3 Compliance with study intervention

We assess the auto-declared compliance of the patients in the intervention (decolonization) arm via questionnaires and by recuperating the empty packages of the decolonization sets.

8.4 Data Collection and Follow-up for withdrawn participants

Withdrawn participants are instructed to continue surveillance according to the protocol and to contact the investigators if there are any questions or concerns that arise after completing the study.

8.5 Trial specific preventive measures

n.a.

8.6 Concomitant Interventions (treatments)

Standard wound control for all patients will include eventual wound debridement (during hospitalization or at clinic visits and only if clinically indicated), regular wound care with dressing changes and eventual VAC use in selected cases upon surgical indication. We will avoid topical antibiotics.

8.7 Accountability of the investigational product

We recuperate the packages of the used decolonization sets from the patients during hospitalization and/or shortly after surgery and this process will be documented (e.g. accountability log). This log should also serve to record any damages of the set.

8.9 Return or Destruction of Investigational Product

Returned used and unused decolonization sets will be destroyed at the end of the investigation, as the sets will be provided for the purpose of this clinical investigation only. The patient will also have be informed about the return of the investigational product. This notice will be captured in the participant information sheet or in the informed consent.

9. STUDY ASSESSMENTS

9.1 Assessment of primary outcome

Judged by local wound healing (healed vs delayed wound healing according to Reference[4]). The wound healing will be assessed visually by orthopaedic surgeons. Wound nurses and the study investigators may help in the assessment of wound problems, but in case of doubtful cases, the opinion of the treating physician will dominate the decision upon the presence or absence of wound problems and/or infection.

Persistent postsurgical pain, serum laboratory markers, or the patient’s subjective wound feelings will not serve as objective criteria for wound healing (or in the contrary, wound discharge and dehiscence). A wound problem solely due to stitches and localized suture problems will not count as a wound problem. A keloid or altered pigment features will not be counted as a problem in this trial. Remission is defined as the absence of any problems and as the absence of any clinical signs of infection.

The primary outcome will be assessed on both, the Intention-to-treat and the per-protocol populations.

9.1.2 Assessment of other outcomes of interest

Secondary objectives are adverse events, unplanned revision surgery for non-infection problems in same time period, and the subjective opinion on the decolonization (only for patients being decolonized). We will note them during the study visits and per questionnaires (for the patients participating in the decolonization.

9.1.3 Assessment of safety outcomes

9.1.3.1 Adverse events

See chapter 10

9.1.3.2 Laboratory parameters

n/a. No study specific laboratory parameters will be assessed

9.1.3.3 Vital signs

n/a. No study specific vital sign measurements will be performed. These vital signs are however col-

lected and noted several times per day according to the clinical protocols of the operated patients for various reasons. We will not add additional criteria in terms of the vital signs only for the sake of our trial.

9.1.4 Assessments in participants who prematurely stop the study

Participants who prematurely stop the investigation are defined as patients who withdraw consent or who, in the opinion of the investigator, are no longer eligible to participate in the study. Where possible, such patients will complete an early-termination visit to undergo all assessments applicable to the corresponding (or next) scheduled study visit. For these patients, we will record eventual adverse events, physical examination, laboratory parameters, vital signs during a follow-up period determined by clinical control, which is usually up to one-year post intervention.

9.2 Procedures at each visit for both prospective-randomized studies

At enrollment (Visit 1 / Day 1), patients will be randomized at ratio 1:1 into the investigational group (decololization) and the control group (no decolonization). The study investigators will instruct the correct decolonization procedure. During the decolonization period, patients can also review the patient information leaflet of the set for details on the decolonization procedure. The questionnaire will be handed over to the decolonization patients.

The treatment period includes the following study visits:

- Visit 1 - Enrollment (Day 1)
- Visit 2 - End-of-Treatment (EOT) -Visit is the Day of Surgery or Day 1-2 after surgery
- Visit 3 - Test-of-Cure-Visit (TOC) (clinical surgical control) - Day 42 (+/- 14 days)
- Follow-up (eventually by phone call) for implant-related surgery after 1 year (+/- 2 months).

During the study visits, we assess the history, wounds, adverse events, and the functional status. We examine the patients according to the orthopedic standard, and add supplementary laboratory exams and radiology, only if clinically indicated.

9.2.1 Enrolment (Visit 1)

Information collected during routine pre-surgical consultation and during orthopaedic surgery is not study specific. This data will be used as general demographic information and medical history within the study in case of an orthopaedic infection and study participation.

If a patient appears to be eligible the following study-related procedures are performed:

1. Patient information and obtaining written informed consent.
2. Assign a study identification number.
3. Record/complete medical history and demographics.
4. Review inclusion/exclusion criteria.
5. Randomize the patient and handing out of the decolonization set.

9.2.2 Visit 2 (End-of-Treatment)

1. Record any concomitant medications as well as any additional interventions required
2. Adaptation of (empirical) antibiotic therapy, if not done before.
3. Assess all adverse events of therapy.

9.2.3 Visit 3 (Test-of-Cure)

Every effort will be made to ensure that final efficacy assessments (i.e., primary outcome data) are available for all subjects. As all our study visits are part of the routine surgical and postsurgical assessments, we do not expect major drop-out rates; or drop out in very exceptional situations (e.g. transfer to another service because of stroke, non-infectious postsurgical fatalities, etc.) Outpatients should return to the clinic (assessments can be performed in the hospital for inpatients), where the following assessments will be performed:

1. Assess anamnestically all past adverse events of surgery, the hospitalization and decolonization
2. Record clinical and microbiological infection and its treatment (if any)

9.2.4 Follow-up visit at approximatively 1 year (for implant-related orthopaedic surgery)

3. Anamnestic assessment of all past adverse events of surgery, the hospitalization and decolonization

Record of clinical and microbiological infection and its treatment (if any).

9.2.5 Early Termination of Study Patients

Patients who withdraw consent or who, in the opinion of the investigator, are no longer able or eligible to participate in the study (including patients who require antibiotic therapy beyond EOT) will be early terminated. Where possible, such patients will complete an early-termination visit to undergo all assessments applicable to the corresponding (or next) scheduled study visit.

10. SAFETY

10.1 Decolonization Set

During the study, all serious adverse events (SAEs) are collected, fully investigated and documented in source documents and case report forms (CRF). Study duration encompasses the time from when the participant signs the informed consent until the last protocol-specific procedure has been completed, including a safety follow-up period. This is 42±14 days, or 1 year in case of an orthopaedic implant.

10.1.1 Treatment by specialists

All surgeries will be performed in the supervision and participation of an advanced and experienced surgeon. The decolonization is ordered and supervised by the study investigators with professional experience in the prevention of orthopedic SSIs. The current medications of the operated study patients, as well as possible interactions, will be controlled by the Head of Pharmacy at the Balgrist.

10.1.2 Definition and assessment of (serious) adverse events and other safety related events

Adverse event

An **Adverse Event (AE)** is any untoward medical occurrence in a patient or a clinical investigation participant administered the investigational product and which does not necessarily have a causal relationship with the investigational product. An AE can therefore be any unfavourable and unintended sign (including an abnormal laboratory finding), symptom, or disease temporally associated with the use of the investigational product, whether or not related to the investigational product.

Assessment of Causality

The investigators will make a causality assessment of the event in relation to the investigational product, based on the criteria listed in the ICH E2A guidelines:

Relationship	Description
Definitely	Temporal relationship<br>Improvement after dechallenge*<br>Recurrence after rechallenge<br>(or other proof of drug cause)
Probably	Temporal relationship<br>Improvement after dechallenge<br>No other cause evident
Possibly	Temporal relationship<br>Other cause possible
Unlikely	Any assessable reaction that does not fulfil the above conditions
Not related	Causal relationship can be ruled out
*Improvement after dechallenge only taken into consideration, if applicable to reaction	

Adverse device effect (ADE, device or investigational product related)

An ADE is any AE for which the causality assessment of the event in relation to the investigational product is possible, probable or definite.

A **Serious Adverse Event (SAE)** is classified as any untoward medical occurrence that:

- results in death,
- is life-threatening,
- requires in-patient hospitalization or prolongation of existing hospitalisation,
- results in persistent or significant disability/incapacity, or
- is a congenital anomaly/birth defect.

In addition, important medical events that may not be immediately life-threatening or result in death, or require hospitalisation, but may jeopardise the patient or may require intervention to prevent one of the other outcomes listed above should also usually be considered serious.

Examples of such events are intensive treatment in an emergency room or at home for allergic bronchospasm, blood dyscrasias or convulsions that do not result in hospitalization, or development of drug dependency or abuse.

SAEs will be followed until resolution or stabilisation. Participants with ongoing SAEs at study termination (including safety visit) will be further followed up until recovery or until stabilisation of the disease after termination.

So far, no serious adverse events as well as adverse events in relation to octenisan® md nasal gel and octenisan® washlotion have been observed in clinical studies.

Unexpected Serious Adverse Drug Reaction

An "unexpected" adverse drug reaction is an adverse reaction, the nature or severity of which is not consistent with the applicable product information (e.g. Investigator's Brochure for drugs that are not yet approved and Product Information for approved drugs, respectively).

Suspected Unexpected Serious Adverse Reactions (SUSARs)

The Sponsor-Investigator evaluates any SAE that has been reported regarding seriousness, causality and expectedness. If the event is related to the investigational product and is both serious and unexpected, it is classified as a SUSAR.

Assessment of Severity

This study uses a severity grading scale as described in the "Common Terminology Criteria for Adverse Events CTCAE Version 4.

10.1.3 Reporting of serious adverse events (SAE) and other safety related events

Reporting of SAEs

All SAEs must be reported immediately and within a maximum of <u>24 hours</u> to the Sponsor-Investigator of the study. The Sponsor-Investigator will re-evaluate the SAE and return the form to the site.

SAEs resulting in death are reported to the local Ethics Committee (via local Investigator) <u>within 7 days</u>.

Equally, the donator schülke will be informed in the event of any of the below in section 10.1.3. The information to schülke will be entirely anonymous.

Reporting of SUSARs

A SUSAR needs to be reported to the local Ethics Committee (local event via local Investigator) <u>within 7 days</u>, if the event is fatal, or <u>within 15 days</u> (all other events).

Reporting of Safety Signals

All suspected new risks and relevant new aspects of known adverse reactions that require safety-related measures, i.e. so-called safety signals, will be reported to the Sponsor-Investigator within 24 hours. The Sponsor-Investigator will report the safety signals within 7 days to the local Ethics Committee (local event via local Investigator).

Reporting and Handling of Pregnancies

This study, all antibiotics and therapeutic surgeries, have no specific relation to pregnant or breastfeeding women and their children. However, for purely formality reasons, we will exclude pregnant and/or breastfeeding women..

Any pregnancy during the treatment phase of the study and within 30 days after discontinuation of study medication will be reported to the Sponsor-Investigator within 24 hours. The course and outcome of the pregnancy will be followed up carefully, and any abnormal outcome regarding the mother or the child should be documented and reported.

Periodic reporting of safety

An annual safety report on the participant is submitted once a year to the local Ethics Committee via the Lead Investigator. We, moreover, will perform statistical interim (futility) analysis on an annual basis.

10.1.4 Follow up of (Serious) Adverse Events

Participants terminating the study (either regularly or prematurely) with reported ongoing SAE, or any ongoing AEs of laboratory values or of vital signs being beyond the alert limit will return for a follow-up investigation. This visit will take place up to 30 days after terminating the treatment period. Follow-up information on the outcome will be recorded on the respective AE page in the CRF/eCRF. All other information has to be documented in the source documents. Source data have to be available upon request.

In case of participants are lost to follow-up, efforts will be made and documented to contact the participant to encourage him/her to continue study participation as scheduled. In case of minor AE, a telephone call to the participants is acceptable.

All new SAE or pregnancies that the investigators will be notified of within 30 days after discontinuation of investigational product will be reported in appropriate report forms and in the CRF/eCRF if required.

Follow-up investigations may also be necessary according to the investigator’s medical judgment even if the participant has no AE at the end of the study. However, information related to these investigations does not have to be documented in the CRF/eCRF, but must be noted in the source documents.

11. STATISTICAL METHODS

11.1 Main hypotheses

Among our selected study participants with an elevated risk for SSI and wound revisions, the presurgical decolonisation may reduce surgical revisions by 5% (from 10% without decolonization to 5 % with decolonization) as compared to the untreated patient group.

11.2 Determination of Sample Size

In our hospital, postoperative wound problems occur in at least 5% percent of all interventions. Incidence for both, revision surgery and/or SSI, in our selected patient population is set for 10% according to our clinical experience among the selected patient population that composes our study population.

We perform a superiority RCT with a power of 80% in favor of the decolonization. With 95% event-free surgeries in the decolonization arm versus 90% in the standard arm, we formally need 2 x 474 orthopedic surgery episodes, which we sum up to 2 x 500 surgeries (n = 1’000). A patient can be included several times as long as he/she gets not infected and has not witnessed postoperative wound problems.

11.3 Planned Analyses

All analyses will be performed for the entire study population. In a second step, all analyses will be separately performed according substrata of patients, based on the type of orthopaedic surgical procedure (e.g. arthroplasties, implant-related surgeries, etc.). We will use descriptive statistics and will perform group comparisons (using the Pearson-χ^2-test, the Fischer-exact-test, or the Wilcoxon-ranksum-test), as appropriate. We will also perform composite (SSI and wound problems) and separate (SSI or wound problems) multivariate analysis using a Cox regression model targeting the co-primary outcome variables. For the primary outcome parameter, univariable and multivariable results will be computed using a Cox regression analysis. Variables with a p value ≤0.2 in univariate analysis will be included in a stepwise forward selection process for multivariate analysis. Key variables will be checked for co-linearity and interaction. The number of variables in the final model is limited to the ratio of 1 variable to 5 to 8 outcome events[14]. The significance level is p<=0.05 (two-tailed).

11.3.1 Interim analysis and early termination

We will perform one interim analysis one year (+/- 2 months) after the inclusion of the first patient. If group comparison between the decolonization and non-decolonization arms are striking and statistically significant in terms of any study objectives, the independent Data Monitoring committee will decide upon the interruption and early termination of the trial. Otherwise, the study continues. Of note, the Data Monitoring committee has the right to call on a premature, additional interim analysis.

If the crude group comparison analysis is not sufficiently meaningful, we will perform a futility analysis to check if the expected statistical power for the final analysis will not be < 30% and therefore unacceptable. If it is lower than 30%, we will consider the trial will not be able to demonstrate the result, and the recruitment will be no more ethical.[15] The most frequents conditional power evaluated under the current trend (i.e. using the information from the collected data) will be assessed. It was demonstrated that futility analyses decrease the statistical power of the final analysis in superiority trials, but in our knowledge, this topic was not explored for non-inferiority trials. To balance (at least partially) this loss of power, we may recruit 100 supplementary patients per arm. E.g. approximately 600 episodes in each randomization arm.

11.3.2 Final analyses

The intent-to-treat (ITT) population will consist of all randomized patients. Patients will be analysed according to treatment group assignment. Patient disposition and baseline characteristics will be based on the ITT population.

The per-protocol (PP) population will consist of all randomized patients who complete the study (or who are otherwise defined as a treatment failure) according to the clinical investigation plan and who have not deviated significantly from the protocol. All efficacy analyses will be repeated using the PP population. Any analysis involving microbiological assessments will exclude patients without an assigned baseline pathogen.

11.4 Handling of missing data and drop-outs

Missing data regarding the outcome parameters and the decolonization will lead to patient dropout of the study. Drop-outs will be reported in the patients & methods section of the publication, drop-out data will archived for a minimum of 10 years after study termination or in case of premature termination of the clinical trial. Major deviations from the protocol will be regarded as drop-outs and excluded from both, the ITT and the PP populations. The independent Data Monitoring Committee may help in case of difficult interpretations of the case among the trial investigators.

12. QUALITY ASSURANCE AND CONTROL

The Sponsor-Investigator will implement and maintain quality assurance and quality control systems with written SOPs and Working Instructions to ensure that the clinical investigation is conducted, and data are generated, documented (record), and reported in compliance with the clinical investigation plan, GCP, and applicable regulatory requirement(s). Monitoring and audits will be conducted during the the study for quality assurance purposes.

12.1 Data handling and record keeping / archiving

Data is exclusively stored using the secured REDCap® electronic data capture tool. The PI is responsible for collection of data and possesses the screening log, where confidentiality is ensured by using participants' ID. Study IDs are distributed by REDCap® automatically in ascending order. Access authorization via Log-in (User) and password will be given by the PI to people on the staff list (people involved in the study), as necessary. For this reason, data cannot be changed by non-authorized people. REDCap® documents every relevant processing step to ensure traceability with registration software and is secured daily via backups. All transaction logs between performing of two backups will be secured for one week while every study data in REDCap® will be secured for an unconfined time, at least for 10 years. Its data base server is allocated in highly modern rooms in Rümlang ZH and Altstetten ZH with protection of access. Collected data of this study is visible for inspection of independent ethic committee and authorities.

When the study is terminated, data will be stored in the same system. Data can only be accessed by defined persons that have contributed to the project. Source Data are going to be stored in the institutions PACS and KISIM system according to the institutional standard at the Balgrist.

12.1.1 Case Report Forms

Electronic case report forms (eCRF) will be used, one for each enrolled study participant, to be filled in with all relevant data pertaining to the participant during the study. All participants who either completed the screening successfully or were considered not-eligible or were eligible but not enrolled into the study have to be documented on a screening log. The participation of each study participant will be documented on the Enrolment Log. For data and query management, monitoring, reporting and coding an internet-based secure data base REDCap® developed in agreement to the Good Clinical Practice (GCP) guidelines will be used. It is the responsibility of the PI to assure that all data in the course of the study will be entered completely and correctly in the respective data base. Corrections in the eCRF may only be done by the investigator or by other authorized persons. In case of corrections the original data entries will be archived in the system and can be made visible. For all data entries and corrections date, time of day and person who is performing the entries will be generated automatically. Documented medical histories and narrative statements relative to the participant's progress during the study will be maintained. These records will also include the following: originals or copies of laboratory and other medical test results (e.g. ECGs, etc.) which must be kept on file with the individual participant's eCRF. The investigators perform a complete and accurate documentation of the participant data in the eCRF.

12.1.2 Specification of source documents

Source data will be available at the site to document the existence of the study participants and substantiate the integrity of study data collected. Source data will include the documents relating to the study, as well as the medical treatment and medical history of the participant.

The following information (at least but not limited to) will be included in the source documents:

- Demographic data (age, sex).
- Inclusion and Exclusion Criteria details.
- Participation in study and signed and dated Informed Consent Forms.
- Visit dates.
- Medical history and physical examination details.
- Key efficacy and safety data (as specified in the protocol).
- SAEs, AEs and concomitant medication.
- Results of relevant examinations.
- Laboratory printouts.
- Reason for premature discontinuation.
- Randomization number.

Source data will always be kept with the regular patient file.

The following documents are also considered source data, including but not limited to:

- SAE worksheets.
- Nurse records, records of clinical coordinators.
- Medical records from other department(s), or other hospital(s), or discharge letters and correspondence with other departments/hospitals, if participant visited any during the study period and the post study period.

12.1.3 Record keeping / archiving

All study data must be archived for a minimum of 10 years after study termination or premature termination of the clinical trial. Data are stored using the proprietary hospital information system and REDCap electronic data capture tool hosted at the Balgrist.

12.2 Data Management System, access and back-up

Subject-related data will be stored in the research electronic data capture software REDCap. Back up will be kept on a hard drive belonging to the PI and later on stored in the archives of the UKB, as mentioned above. The PI and the co-investigators are responsible for data recording. The PI will grant the relevant personnel user rights to view and/or edit data entries by password as applicable. All edits will be automatically documented in the change history log.

12.2.1 Analysis and archiving

For data analysis, subject-related data from REDCap will be exported and analyzed in statistics software (IBM – SPSS and/or STATA, Version 14, College Station, USA). Before data export, all patient identifiers will be removed. All eCRF data will be stored for a minimum of 20 years.

12.3 Monitoring

Regular monitoring visits at the investigator's site prior to the start, during the interim analysis and at the end of the study, will help to follow up the progress of the clinical study, to assure utmost accuracy of the data and to detect possible errors at an early time point. The Sponsor-Investigator organizes professional independent monitoring for the study.

All original data including all patient files, progress notes and copies of laboratory and medical test results must be available for monitoring. The monitor will review all or a part of the eCRFs and written informed consents. The accuracy of the data will be verified by reviewing the above referenced documents.

One monitoring visit at the investigator's site prior to the start and twice during the course of the study will be organised by the Sponsor-Investigator. Furthermore, there will be a close-out visit at the study end. During the monitoring, all documents including source data/documents will be accessible for the monitor and all questions will be answered.

**Study period**	**Time**	**Monitoring**
Before study	January or February 2023	Monitoring will be informed about study conduct concerning data sampling and safety reporting.<br>Monitor controls if<br>• Documents are approved<br>• Documents are at site<br>• Investigators are familiar with study protocol and safety reporting<br>• Investigators know their duties and responsibilities
During Study	Spring 2024	All subjects: SDV for existence and informed consent<br>First trial participant and at least 10% of trial participants recruited at the time of the monitoring visit, as far as available: eligibility, primary endpoint, SAEs
Study end	December 2024<br>January 2025	Control for completeness of source data

12.4 Audits and Inspections

A quality assurance audit/inspection of this study may be conducted by the competent authority or CEC, respectively. The quality assurance auditor/inspector will have access to all medical records, the investigator's study related files and correspondence, and the informed consent documentation that is relevant to this clinical study. The investigator will allow the persons being responsible for the audit or

the inspection to have access to the source data/ documents and to answer any questions arising. All involved parties will keep the patient data strictly confidential.

12.5 Confidentiality, Data Protection

Direct access to source data may be granted in the case of monitoring, audit or inspections. All personnel must treat patient data as confidential. As far as possible, encoded data will be used. Only persons listed on the staff list have access to the source data.

12.6 Storage of biological material and related health data

All health-related patient data will be stored and archived in the data capture software REDCap. Patient-source data will be registered using subject identifiers. After full data analysis, all subject identifiers will be erased. Patient-source data may still be saved in the patient's medical record. Collection, disclosure, storage of patient-related data is carried out in accordance with Swiss data protection regulations and the Human Research Act. A requirement is the informed consent of every subject prior to inclusion in the clinical trial.

13. PUBLICATION AND DISSEMINATION POLICY

After the statistical analysis of this trial, the sponsor-investigator will make every endeavour to publish the data in (a) medical journal(s), to be able to communicate the results to healthcare professionals, the public and other relevant groups. All participants will be sent a free copy of the published article. There will not be any publication restriction and we plan to sort at least three major publications. We will also present preliminary results in national, regional, and international scientific meetings.

All investigators indicated in this protocol, and eventually additional colleagues participating in the future, will be co-authors of this study according to their individual contributions. The main study nurse, all Sponsors and Principal Investigators will participate in all publications. In selected publications, members of the corresponding orthopaedic teams will participate as co-authors and depending on their investment into the study.

14. FUNDING, BUDGET AND SUPPORT

The global (undetailed) Budget is planned as follows (all currencies in Swiss Francs CHF):

**Costs**	Total	1st Year	2nd Year
a. Ethical Comittee	3,000	3,000	0
b. Monitoring	5,000	2,500	2,500
c. Nurse 20% for 2 years	39,000	19,500	19,500
d. Social securities	5,654	2,827	2,827
e. Miscellaneous	2,346	423	1,923
**Swiss Francs (CHF)**	**55,000**	**28,250**	**26,750**

Schülke & Mayr AG, Hungerbüelstrasse 22, 8500 Frauenfeld, Schweiz will support the study with an unconditional donation of 55,000.- (fifty-five thousand) Swiss Francs (paid in two tranches), which will be used for the salary of the study / infection control nurses, the Monitoring and for the Ethical Committee fees. The study team will not make financial profit and cover the minimal operational costs.

Schülke & Mayr GmbH will also grant/donate all the decolonization kits used for the study, for free (including the corresponding transport costs).

Schülke & Mayr GmbH has the rights to consult the data and the draft, but will not be part of the study / academic team that will publish the results of the BALGDEC study.

14.1. Payments

Installments will be paid by February 2023 to a Balgrist funding account:

- By February 2023, CHF 47.500.- (forty-seven thousand five hundred Swiss Francs) by Schülke & Mayr AG, Hungerbüelstrasse 22, 8500 Frauenfeld, Schweiz

  In 2024 (study ending) the remaining CHF 7.500.- (seven thousand five hundred Swiss Francs) will be paid to the Institution's account by Schülke & Mayr AG, Hungerbüelstrasse 22, 8500 Frauenfeld, Schweiz.

The 550 sets (500 for the study, 50 in reserve) are provided by Schülke Switzerland

15. INSURANCE

The standard Balgrist research insurance is applicable.

Insurance police Nr. 14.050.565 Winterthur Versicherung.

Any damage developed in relation to study participation is covered by this insurance. So as not to forfeit their insurance cover, the participants themselves must strictly follow the instructions of the study personnel. Participants must not be involved in any other medical treatment without permission of the principal investigator (emergency excluded). Medical emergency treatment must be reported immediately to the investigator. The investigator must also be informed instantly, in the event of health problems or other damages during or after the course of study treatment. The investigator will allow delegates of the insurance company to have access to the source data/documents as necessary to clarify a case of damage related to study participation. All involved parties will keep the patient data strictly confidential. A copy of the insurance certificate will be placed in the Investigator’s Site File and the trial master file.

16. REFERENCES

1) Uçkay I, Hoffmeyer P, Lew D, Pittet D. Prevention of surgical site infections in orthopaedic surgery and bone trauma: state-of-the-art update. J Hosp Infect. 2013 May;84(1):5-12.

2) Tsang STJ, McHugh MP, Guerendiain D, et al. Evaluation of Staphylococcus aureus eradication therapy in orthopaedic surgery. J Med Microbiol. 2018 Jun;67(6):893-901.

3) Dancer SJ, Christison F, Eslami A, et al. Is it worth screening elective orthopaedic patients for carriage of Staphylococcus aureus? A part-retrospective case-control study in a Scottish hospital. BMJ Open. 2016 Sep 6;6(9):e011642.

4) World Health Organization. Global guidelines for the prevention of surgical site infection, 2nd ed. https://www.who.int/publications/i/item/global-guidelines-for-the-prevention-of-surgical-site-infection-2nd-ed.

5) Prentice HA, Chan PH, Champsi JH, et al. Temporal Trends in Deep Surgical Site Infections After Six Orthopaedic Procedures Over a 12-year Period Within a US-based Healthcare System. J Am Acad Orthop Surg. 2022 Sep 7.

6) Rohrer F, Nötzli H, Risch L, et al. Does Preoperative Decolonization Reduce Surgical Site Infections in Elective Orthopaedic Surgery? A Prospective Randomized Controlled Trial. Clin Orthop Relat Res. 2020 Aug;478(8):1790-1800.

7) Schülke & Mayr GmbH GmbH. Prävention schlägt Infektion! https://www.schuelke.com/wMedia/docs/folder-broschueren/infektionspraevention/schuelke_Praevention-schlaegt-Infektion_Folder-2021.pdf

8) https://www.bio-apo.ch/product/octenisan-set-waschlotion-nasengel.2157023.html?p=16&gclid=CjwKCAjwsfuYBhAZEiwA5a6CDGLusE-Ms5yWCFJl-skkkrchWl6WQ9DPK491JnDMJqLJuLPe-u-ZhRoCcTAQAvD_BwE

9) Uçkay I, Harbarth S, Ferry T, et al. Methicillin-resistance in orthopaedic coagulase-negative staphylococcal infections. J Hosp Infect. 2011 Nov;79(3):248-53.

10) Cohen ME, Salmasian H, Li J, et al. Surgical Antibiotic Prophylaxis and Risk for Postoperative Antibiotic-Resistant Infections. J Am Coll Surg. 2017;225(5):631-8.

11) Uçkay I, Agostinho A, Belaieff W, et al. Noninfectious wound complications in clean surgery: epidemiology, risk factors, and association with antibiotic use. World J Surg. 2011; 35(5):973-80.

12) Vuichard-Gysin D, Senn L, Tschudin-Sutter S, et al. Swiss-NOSO: Prävention und Kontrolle von multiresistenten Erregern (MRE) im Nicht-Ausbruch-Setting. Version 1.0, Oktober 2021. https://www.swissnoso.ch/fileadmin/swissnoso/Dokumente/5_Forschung_und_Entwicklung/8_Swissnoso_Publikationen/211115_StAR_Teil_II_DE_MDRO-non-outbreak_FINAL.pdf.

13) Karaca S, Çikirikcioğlu M, Uçkay I, et al. Comparison of vacuum-assisted closure device and conservative treatment for fasciotomy wound healing in ischemia-reperfusion syndrome: preliminary results. Int Wound J. 2011; 8(3):229-36.

14) Vittinghoff E, McCulloch CE. Relaxing the rule of ten events per variable in logistic and Cox regression. Am J Epidemiol. 2007;165:710-18.

15) Snapinn S, Chen MG, Jiang Q, Koutsoukos T. Assessment of futility in clinical trials. Pharmaceutical Statistics. 2006;5:273-81.

Appendix 1 Decolonization Set – Manufacturer’s brochure. See separate file

Appendix 2 Questionnaire in German language. See separate file
