# Supplementary material for: General skin and nasal decolonization with octenisan® set before and after elective orthopedic surgery in selected patients at elevated risk for revision surgery and surgical site infections—a single-center, unblinded, superiority, randomized controlled trial (BALGDEC trial)
Source: Trials. 2024 Jul 8;25:461. doi: 10.1186/s13063-024-08173-y (PMC11229206; doi:10.1186/s13063-024-08173-y)
Supplement: Supplementary file 2 — Supplementary Material 2: Supplementary file 2. Model consent form. [file 13063_2024_8173_MOESM2_ESM.docx]

**Patient information**

**Skin and nose decolonization prior to orthopaedic surgery to reduce the risk of postoperative infections and wound healing disorders**

*Original title: General Skin and Nasal Decolonization with Octenisan® Set before and after Elective Orthopedic Surgery in Selected Patients at Elevated Risk for Revision Surgery and Surgical Site Infections–a Single-Center, Unblinded, Superiority, Randomized-Controlled Trial*

This study is organized by the Investigator Team: Prof. Dr. med. Ilker Uçkay, Dr. Ines Unterfrauner, Ms. Nadja Bragatto-Hess, Mr. Thorsten Studhalter, Balgrist University Hospital.

Dear Patient, Dear Patient

You will soon have an orthopedic surgical intervention. We invite you to participate in this study on the prevention of postoperative infections and associated wound problems.

**1. Aim of the study**

We investigate whether a preoperative skin and nose decolonization, with octenidin for five days, can reduce the risk of postoperative infections and of the associated wound problems.

**2. Inclusion of patients**

All adult persons with imminent orthopedic surgery who have an increased risk of wound problems and wound infections can participate. You must also be at least 18 years old. Persons who are already undergoing surgery for any infection, who reveal a diabetic foot syndrome, a body mass index of more than 35 kg/m^2^, or without increased infection risks, cannot participate.

**3. General information**

The human skin surface is naturally colonized with bacteria. Upon surgery, these bacteria can get into the open wound and contribute to a postoperative infection. Theoretically, a “decolonization” procedure might reduce the density and the number of these skin bacteria immediately before surgery. This presurgical decolonization is often recommended in medical guidelines. But not all studies showed an advantage, especially if this decolonization is performed in all orthopedic patients without distinction of the patients. Studies targeted vulnerable patients only (patients with an increased risk of infection), or studies on the decolonization procedure using a commercial set, exist only rudimentarily.

We perform such a study with the help of a prefabricated decolonization set containing the antiseptic substance octenidin. The substance and the set are freely available on the Swiss market. This study has no additional costs for you and is partially financed with a grant from Schülke & Mayr AG. The company has no insight into your personal data.

**4. Procedure**

If you participate in this study, you will be randomly assigned either to the decolonization group, or the non-decolonization group. The probability of the attribution is the same. The patients in the decolonization group receive the set and apply it daily, during five days, before the scheduled surgery. Specifically, the use the octenisan^®^ wash lotion once a day; and the octenisan^®^ nasal gel 2-3 times a day. If you are in the decolonization group, you will also answer a short questionnaire about the decolonization procedure. Everything else, such as the surgery or the perioperative antibiotic prophylaxis, remains the same. While decolonizing, please do not use any other lotions or antiseptic creams. The use of a perfume is allowed. In the other, non-decolonized group, no products are applied for decolonization. Members of this group do not have to answer questionnaires. We only analyze their medical and nursing data related to the scheduled surgery. You may be excluded from the study in your best interest. This can happen if, from a medical point of view, you do not qualify to participate.

**5. Benefits**

You might benefit from decolonization (e.g. less wound healing disorders and less wound infections). However, it may also be that you do not benefit from participating. The anonymized results of this study help future patients.

**6. Rights**

Your participation is voluntarily. If you do not want to participate, you do not have to justify. You may ask questions about the study at any time. To do so, please contact the person named at the end of this information sheet. If you do not participate in this study, you will not be disadvantaged for your further medical and surgical care. The same applies if you revoke your consent at a later date.

**7. Obligations**

As a participant, it is important that you

- adhere to the guidelines and prescriptions of your attending physician.
- Inform your attending physician about the decolonization and report new complaints or changes in your condition.
- inform the investigator about concomitant treatment and therapy with another physicians and surgeons; and about taking medication.
- Answer a short questionnaire (for patients in decolonization arm).
- Return of empty decolonization set boxes during the hospital stay.

**8. Risks and burdens for participants**

The patients who are not decolonized could theoretically have more infections and/or postoperative wound problems. The patients who are decolonized with the set might experience side effects of the products (rash, itching, skin irritation) that are unpredictable.

**9. Other treatment options**

You are not obliged to participate in this study. Then you simply will be not decolonized Everything else remains the same.

**10. Findings from the study**

The investigator will inform you during the study of any new findings that may affect your safety. You will receive the information orally and in written form. You will be informed of these incidental findings that could help to prevent, detect and treat existing or anticipated future diseases. If you do not wish to be informed, please inform your study investigator.

**11. Confidentiality of data and samples**

We will assess your personal and medical data for this study. Very few professionals will see your unencrypted data, and only to perform analyses within the scope of the study. When your data is collected for study purposes, the data is encrypted. Encryption means that all references that could eventually identify you (name, date of birth) are deleted and replaced with a key number. The key list always remains at the Balgrist University Hospital. Those people who do not know the key can therefore not draw any conclusions about your person. In the case of a publication, the summarized data is therefore not traceable to you as an individual. We will not publish your name anywhere, in any report, publication, print or Internet. If raw data has to be made available for the publication of scientific reports in scientific journals, it is always done in encrypted form. Additionally, we may be able to use the data for other research purposes and ask for your signature on the second consent form. This second signature is voluntary and independent of participation in this study.

This study may be reviewed by competent authorities such as an Ethics Committee, *Swissmedic* or the institution that initiated the study. These institutions ensure that the rules are adhered to and that your safety is not compromised. The study leader may need to disclose your personal medical information upon explicit order of these authorities.

**12. Withdrawal**

You can stop and withdraw from the study at any time you wish. The data collected until then will be evaluated in encrypted form. Otherwise, the entire project loses its value. Afterwards, your data remain completely anonymized, i.e. your key assignment will be destroyed so that no one can know that the anonymized data originally came from you.

**13. Compensation for participants**

You will not be remunerated for participating in this study. You, or your health insurance company, will not incur any costs as a result of your participation.

**14. Liability**

If you suffer damage as a result of the study participation, the institution responsible for conducting the study is liable. This procedure is regulated by law. The Balgrist University Hospital engages the insurance AXA Winterthur Versicherungen AG, General-Guisan-Strasse 40, 8400 Winterthur, to be able to pay for liability in the unlikely event of damage. If you have suffered damage, please contact the contact persons mentioned below or the insurance company mentioned above.

**15. Financing of the study**

This study is supported by the company Schülke & Mayr AG with an unconditional grant / donation. Schülke & Mayr AG also donates the decolonization sets for free of charge, and has no insight into its individual and anonymized data.

**16. Contact person**

In case of ambiguities, emergencies, unexpected events, or adverse events related to the decolonization, which occur during or after the study, you can always contact the following investigators:

| Dr. Ines Unterfrauner  Department of Orthopaedics  Balgrist University Hospital  Forchstrasse 340  8008 Zurich  Phone 044 386 11 11  E-Mail: [i nes.unterfrauner@balgrist.ch](mailto:ines.unterfrauner@balgrist.ch) | Prof. Dr. med. Ilker Uçkay  Infectiology, Head of Clinical Research  Balgrist University Hospital  Forchstrasse 340  8008 Zurich  Phone 044 386 11 11  E-Mail: [ilker.uckay@balgrist.ch](mailto:ilker.uckay@balgrist.ch) |
| --- | --- |

**Consent**

**Written declaration of consent to participate in a study project**

Please read this form carefully. Please ask if you do not understand or do not want to know something. Your written consent is required for participation.

| **Title of the study:** | Skin and nose decolonization prior to orthopaedic surgery to reduce the risk of postoperative infections |
| --- | --- |
| **Responsible institution:** | Balgrist University Hospital |
| **Place of implementation**: | Balgrist University Hospital  Forchstrasse 340  CH-8008 Zurich |
| **Responsible investigator at the study site:** | Prof. Dr. med. Ilker Uckay |
| **Participant:** Surname and first name: Date of birth: | female male |

- I was informed orally, and in writing, by the undersigned investigator about the purpose, the course of the project, possible advantages and disadvantages as well as possible risks.
- I participate voluntarily in this project and accept the content of the written information provided on the above-mentioned project. I had enough time to make my decision.
- My questions related to participation in this project have been answered. I keep the written information and receive a copy of my written declaration of consent.
- I am informed that an insurance covers the damage to health, if such occur in the context of the studies.
- I agree that the responsible experts of the project management/client of the study and the ethics committee responsible for this project may inspect my unencrypted data for examination and control purposes, but in strict compliance with confidentiality.
- In the event of study results or incidental findings that directly affect my health, I am informed. If I do not wish this, I will inform my investigator.
- I know that my health and personal data can only be shared in encrypted form for research purposes for this study project.
- I agree that if I continue to be treated outside the study site, the investigator/project leader may contact the treating physicians to request follow-up data relevant to the study.
- I participate in this study voluntarily. I can withdraw from participation at any time and without giving reasons, without having any disadvantages in further medical treatment/care. The data and samples collected until then will still be used for evaluation of the study.
- I am aware that during the study, the requirements and restrictions stated in the patient information must be observed. In the interest of my health, the investigator may exclude me from the study.

| Place, Date | Signature of the participant |
| --- | --- |

**Confirmation of the investigator: I** hereby confirm that I have explained the nature, significance and scope of the study to this participant. I agree to comply with all obligations related to this study in accordance with applicable law. If, at any time during the conduct of the study, I become aware of aspects that could influence the participant's willingness to participate in the study, I will inform him/her immediately.

| Place, Date | Surname and first name Investigator |
| --- | --- |
|  | Signature Investigator |

**Declaration of consent for further use of (genetic) data and biological material in encrypted form (for the further use of data and samples from THIS study)**

| **Participant:** Surname and first name: Date of birth: | female  male |
| --- | --- |

I allow my (genetic) data and samples from this study to be used for medical research. This means that the samples may be stored in a biobank and used for future, as yet undefined research projects for an indefinite period of time. This consent is valid indefinitely.

I decide voluntarily and can revoke this decision at any time. If I resign, my (genetic) data will be anonymized and my samples destroyed. I only inform my investigator and do not have to justify this decision.

I understood that the data and samples are encrypted and the bowl is kept safe. The data and samples can be sent to other data and biobanks in Switzerland and abroad for analysis if they comply with the same standards as in Switzerland. All legal requirements for data protection are complied with.

Normally, all data and samples are evaluated as a whole and the results are published in summary. If there is an important result for my health, it is possible that I will be contacted via my investigator. If I do not wish this, I will inform my investigator.

If results from the data and samples are commercialized, I have no claim to a share of the commercial use.

| Place, Date | Signature of participant (or legal representative) |
| --- | --- |

**Confirmation of the investigator: I** hereby confirm that I have explained to this participant the nature, meaning and scope of the further use of samples and/or genetic data.

| Place, Date | Surname and first name of the informing investigator in block capitals  Signature of the investigator |
| --- | --- |
